# Highly condensed chromatins are formed adjacent to subtelomeric and decondensed silent chromatin in fission yeast

**DOI:** 10.1038/ncomms8753

**Published:** 2015-07-24

**Authors:** Atsushi Matsuda, Yuji Chikashige, Da-Qiao Ding, Chizuru Ohtsuki, Chie Mori, Haruhiko Asakawa, Hiroshi Kimura, Tokuko Haraguchi, Yasushi Hiraoka

**Affiliations:** 1 Advanced ICT Research Institute Kobe, National Institute of Information and Communications Technology, 588-2, Iwaoka, Iwaoka-cho, Kobe 651-2492, Japan; 2 Graduate School of Frontier Biosciences, Osaka University, 1-3 Yamadaoka, Suita 565-0871, Japan; 3 Department of Biological Sciences, Graduate School of Bioscience and Biotechnology, Tokyo Institute of Technology, 4259-B, Nagatsuda, Yokohama 226-8501, Japan

## Abstract

It is generally believed that silent chromatin is condensed and transcriptionally active chromatin is decondensed. However, little is known about the relationship between the condensation levels and gene expression. Here we report the condensation levels of interphase chromatin in the fission yeast *Schizosaccharomyces pombe* examined by super-resolution fluorescence microscopy. Unexpectedly, silent chromatin is less condensed than the euchromatin. Furthermore, the telomeric silent regions are flanked by highly condensed chromatin bodies, or ‘knobs'. Knob regions span ∼50 kb of sequence devoid of methylated histones. Knob condensation is independent of HP1 homologue Swi6 and other gene silencing factors. Disruption of methylation at lysine 36 of histone H3 (H3K36) eliminates knob formation and gene repression at the subtelomeric and adjacent knob regions. Thus, epigenetic marks at H3K36 play crucial roles in the formation of a unique chromatin structure and in gene regulation at those regions in *S. pombe*.

## Abstract (web summary)

The level of chromatin condensation and gene expression is believed to be inversely correlated. Here the authors show that the transcriptionally silent telomere regions are flanked by highly condensed chromatin, and are less condensed than euchromatin in the interphase of *Schizosaccharomyces pombe*.

## Graphical abstract

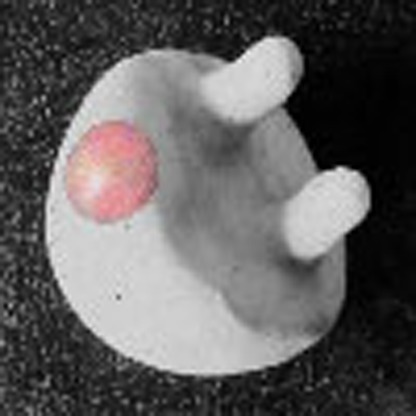

The eukaryotic genome is organized into chromatin domains with distinct structure and function. ‘Euchromatin' is decondensed and capable of gene expression. In contrast, ‘heterochromatin' is condensed and often transcriptionally silent. Foreign genes inserted in the silent regions are stochastically repressed^1^,^2^.

Each chromatin domain is marked up with post-translational histone modifications and histone variants, and such variations in histones play critical roles in the transcriptional regulation of each chromatin region^1^,^2^,^3^,^4^. In general, euchromatin is rich in hyperacetylated histones and heterochromatin is rich in hypoacetylated histones. In the case of methylation, euchromatin is characterized by methylation of histone H3 at lysine 4 (H3K4me1, H3K4me2 and H3K4me3), whereas heterochromatin is characterized by di- and trimethylation of histone H3 at lysine 9 (H3K9me2 and H3K9me3) and, in higher eukaryotes, trimethylation at lysine 27 (H3K27me3). It is known that some histone methylations associate with gene structures in euchromatin—the trimethylation of histone H3 at lysine 4 (H3K4me3) associates with the promoters, while the trimethylation of histone H3 at lysine 36 (H3K36me3) associates with coding regions^4^,^5^.

Studies examining the distribution of histone modifications and DNA-binding proteins suggest that the genomes of *Drosophila* and humans can be classified into five or more distinct chromatin domains^6^,^7^. Despite having accumulated knowledge of chromatin domains at the biochemical level, we know little about the structural organization of chromatin in three-dimensional space. This is because direct observation of small chromatin structures (30–200 nm) has been hindered by the resolution limit of optical microscopes (∼250 nm).

The simple genome of the fission yeast *S. pombe* is a useful model for the study of chromatin. Its small genome (∼12.5 Mb) is organized into only three chromosomes and has a gene density of 0.4 genes per kb, and is thus mostly euchromatin. Well-defined regions of heterochromatin are only found at centromeres, telomeres and mating-type regions. *S. pombe* contains many heterochromatin factors that are conserved in higher eukaryotes, and its nucleosome contains no histone H1 and no known histone H3 variants except for the CENP-A homologue (Cnp1). This makes it one of the simplest model organisms to understand the molecular basis of heterochromatin formation. However, few structural studies have been done of chromatin in *S. pombe* due to its small nucleus (∼2 μm in diameter).

Recent advances in fluorescence microscopy set the new resolution limit beyond the diffraction limit of the light^8^, making it possible to directly observe the chromatin structure in *S. pombe*. We used three-dimensional structured illumination microscopy (3DSIM) that is capable of multicolour, 3D observation at a resolution of 120 and 300 nm in the lateral and vertical directions, respectively^9^,^10^. Here we examined the DNA concentration of chromatin domains marked by histone modifications and other factors. We found that silent chromatin regions had the least condensed chromatin in the interphase nucleus of *S. pombe*. The most condensed region was unexpectedly just next to the subtelomeric silent chromatin, and its condensation is regulated by epigenetic marks at H3K36.

## Results

### The subtelomeric silent chromatin is least condensed

**Figure 1:**
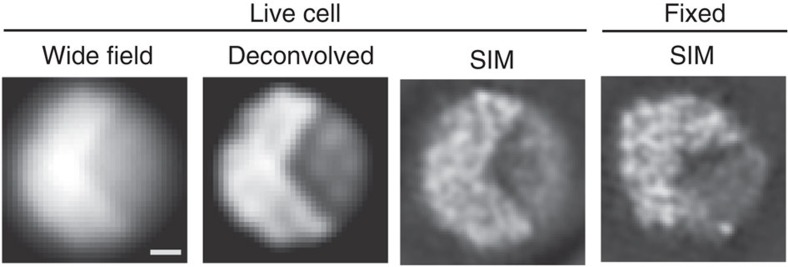
Appearance of chromatin in the interphase nucleus of *S. pombe*. Chromatin structure in the interphase nucleus of *S. pombe* expressing H2B-GFP observed using 3DSIM. Single optical sections are shown. Raw (wide field), deconvolution and 3DSIM images of a single nucleus of a live cell are shown, together with a single 3DSIM image of another fixed cell. The contrast of the images is adjusted to show the background with a very low noise level. Fixed cells showed chromatin structures similar to the live cells. Scale bar, 500 nm.

Interphase nuclei in living *S. pombe* cells have almost uniform chromatin concentration levels when observed with conventional wide-field microscopy or deconvolution (Fig. 1). However, when observed with live 3DSIM, heterogeneous chromatin condensation levels were observed (Fig. 1; also see Supplementary Fig. 1 for the experimental background). We were able to reproduce this chromatin organization after chemical fixation (Fig. 1).

**Figure 2:**
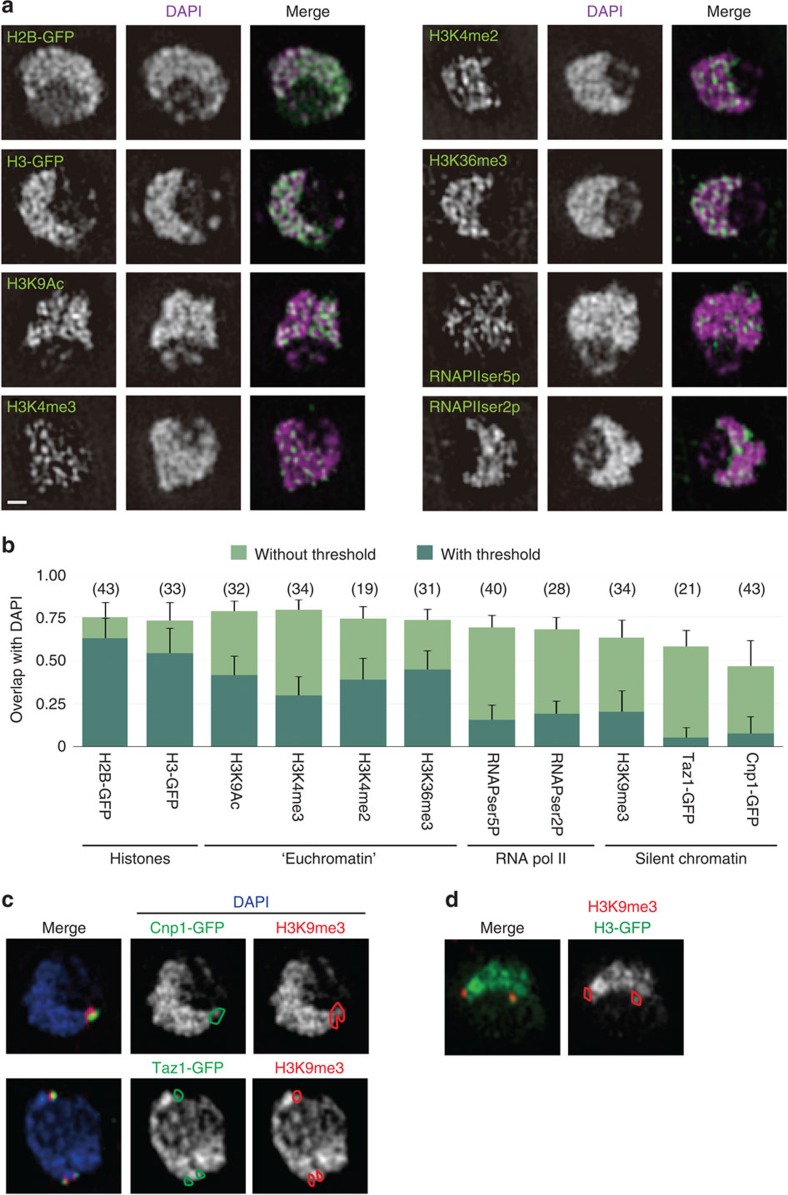
DNA concentration in the interphase nucleus of *S. pombe*. (**a**) Single optical sections obtained with 3DSIM of fixed, immunostained nuclei using antibodies recognizing the indicated epitopes (green) and counterstained with DAPI (magenta). ‘RNAPIIser5p' and ‘RNAPIIser2p' denote RNAPII phosphorylated at serine 5 and 2 of the C-terminal domain, respectively. Scale bar, 500 nm. (**b**) Quantitative representation of the overlap of each chromatin marker with DAPI staining. Bar shows s.d. of nuclei. The value 1.0 indicates perfect overlap of the immunofluorescence with the DAPI fluorescence. Numbers in parenthesis indicate the number of nuclei examined. (**c**) The merged images (left panels) show centromeric (Cnp1-GFP) and telomeric (Taz1-GFP) regions in green and silent loci marked by trimethylation at lysine 9 of histone H3 (H3K9me3) in red, superimposed on DAPI images in blue. The right two columns show the corresponding DAPI images (white) marked with regions of Cnp1-GFP or Taz1-GFP (the green contour) and H3K9me3 regions (the red contour). (**d**) The merged image (left panel) shows H3K9me3 (red) superimposed on the image of histone H3-GFP (green); the right panel shows the corresponding histone H3-GFP image (white) marked with the H3K9me3 region (the red contour).

To analyse which genomic regions were more condensed or decondensed in the interphase nucleus, immunofluorescence of histone modifications and chromatin factors were observed with 3DSIM (Fig. 2a). The chromatin condensation level of each domain was expressed as the DNA concentration measured by 4′,6-diamidino-2-phenylindole (DAPI) staining. Although DAPI has a higher affinity for AT-rich sequences, the GC-content of the genome in *S. pombe* is relatively constant at this resolution. Thus, the immunofluorescence signal overlapping with the DAPI staining should directly reflect the DNA concentration in the target region. The overlap was determined with our original algorithm that measures the fraction of signal that co-localizes with the DAPI signal (see ‘Algorithm for the co-localization analysis' in Methods). Our algorithm allows measurements with or without thresholding. Thresholding has the advantage that it limits the co-localization analysis only to the target signal, but has the disadvantage that it relies on human interpretation of the object. This potential disadvantage is overcome by taking measurements without thresholding, though the difference among data is then modest due to the weak background signal. Here we took measurements using both methods for comparison.

The DNA concentration obtained with histone-GFP (green fluorescent protein), measured as the overlap between DAPI and GFP signals, (Fig. 2b) is assumed to represent the average DNA concentration of all nucleosomes. The DNA concentrations of the regions stained with histone modifications specific to euchromatin (H3K9Ac, H3K4me2/3 and H3K36me3) were similar to each other and to histone-GFP regions. Since the genome of *S. pombe* is mostly euchromatin, these modifications are expected to mark most regions of chromatin. The DNA concentration tended to be lower in regions that were undergoing active transcription by RNA polymerase II (RNAPII), irrespective of its phosphorylation state at the carboxyl (C)-terminal domain (Fig. 2b).

**Figure 3:**
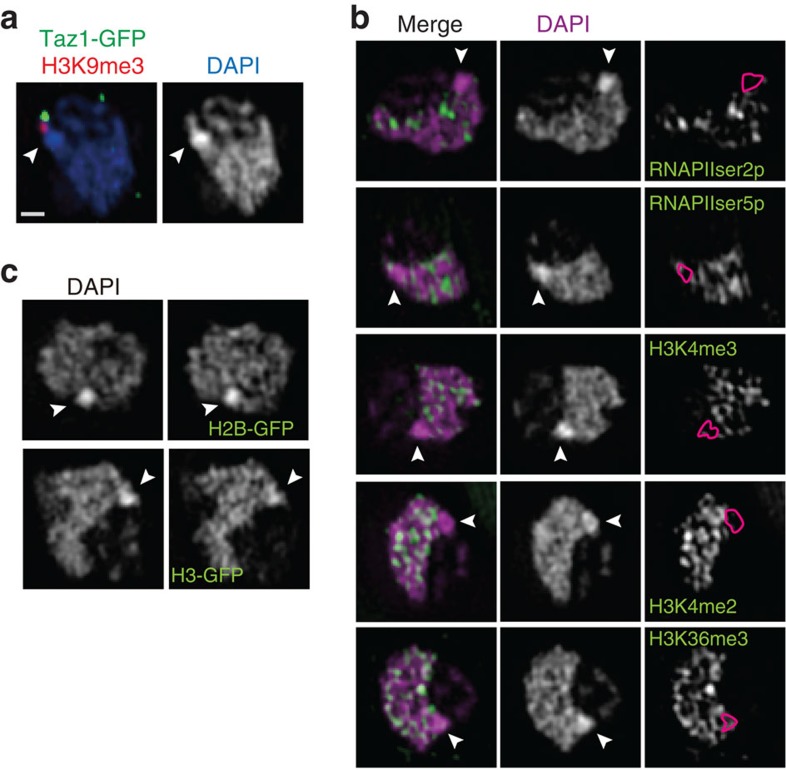
Highly condensed chromatin body devoid of known chromatin markers. Single optical sections with 3DSIM of immunostained nuclei using antibodies recognizing the indicated epitopes. The arrowheads point to the most condensed chromatin body. The bar in (**a**) indicates 500 nm for all panels. (**a**) A region or two of very high DNA concentration (‘knob') stained with DAPI (white in the right panel). In the merged image (left), telomere (Taz1-GFP; green) and silent H3K9me3 foci (red) are superimposed on the DAPI image (blue). (**b**) RNA polymerases or histone modifications are shown in green superimposed on DAPI staining (magenta in the left panels or white in the middle panels). In the right panels, knob regions are indicated by the red contours superimposed on the RNA polymerase or histone modification images as indicated. Markers of euchromatin are absent in knob regions. (**c**) Core histones C-terminally fused with GFP are present in knobs at about the same intensity as DNA stained with DAPI.

**Figure 4:**
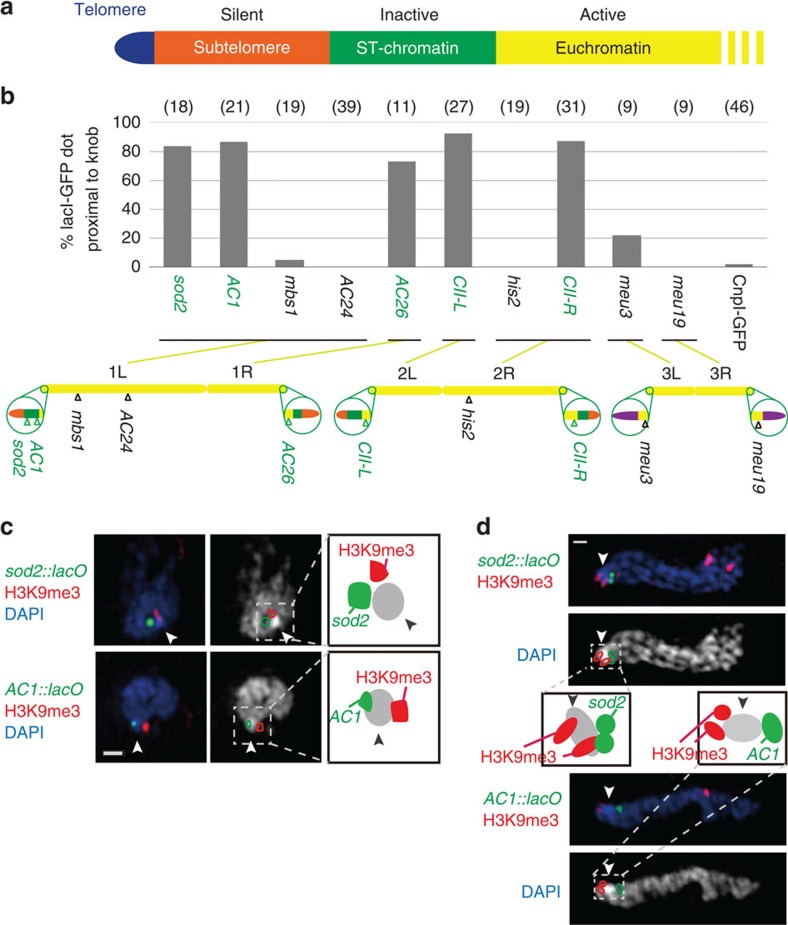
Knobs are formed at ∼50 kb regions adjacent to the subtelomeric silent chromatin of chromosomes 1 and 2. (**a**) A map describing the regions found adjacent to the telomeres of chromosomes 1 or 2. The silent subtelomere and ST-chromatin are ∼50 kb each. (**b**) Percentage of knob-containing nuclei in which the indicated *lacO* loci are proximal (<0.2 μm) to the knobs. Numbers in parenthesis indicate the number of nuclei examined. In the chromosome map, green regions are ‘ST-chromatin', orange regions are subtelomeric silent regions and purple regions are rDNA. (**c**) Two loci (*sod2* and *AC1*) marked with a *lacO* array are found close to knobs, visualized with LacI-GFP (green). DAPI images are shown in blue in the merged image (left). The right panels show the DAPI images (white) superimposed with *lacO*/lacI-GFP and H3K9me3 regions (green and red contours, respectively) relative to the knob region (arrowhead). The positional relationships between the *lacO*/lacI-GFP, H3K9me3 and knob are drawn on the right. Scale bar, 500 nm. (**d**) Knobs in meiotic prophase nuclei. Telomeres are located on the left edge of the nucleus. Labels and drawings are the same as in **c**. Scale bar, 500 nm.

**Figure 5:**
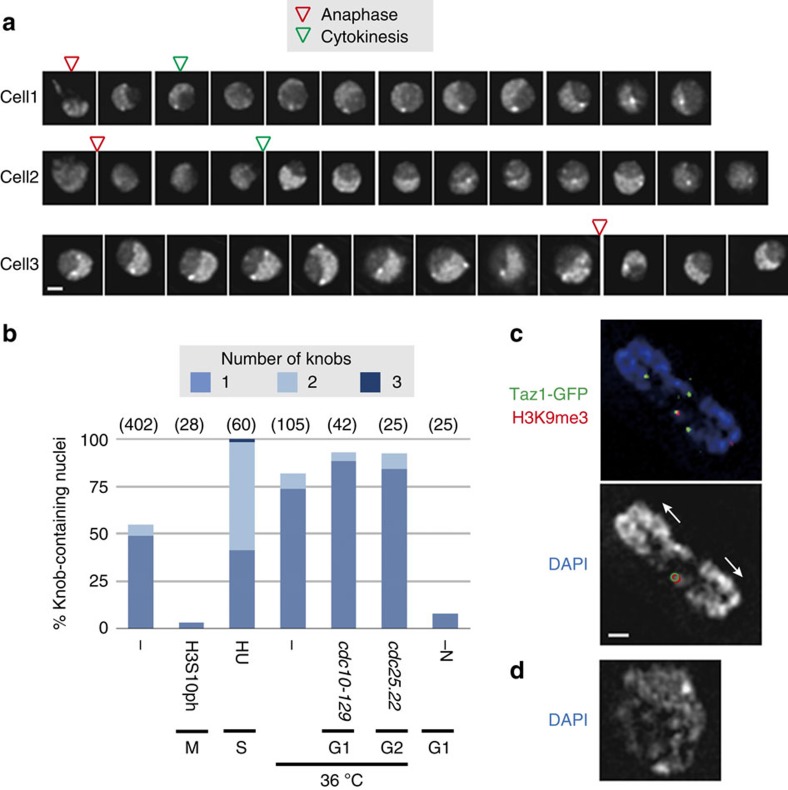
Knob condensation is dynamically regulated in the cell cycle. (**a**) Live imaging of three independent cells expressing H2B-GFP. Pictures were taken every 7.5 min but here images are shown in 15-min steps. Deconvolved optical sections with the brightest dots at each time frame are shown. Scale bar, 1 μm. (**b**) Frequency of knob-containing nuclei. The number of knobs in a nucleus is indicated by different colours. Standard growth conditions (wild type in EMM2 5S at 26 °C) are indicated as ‘−' and any differences from these conditions are indicated. ‘H3S10ph' means standard cells positively stained with anti-H3S10ph antibody, which is specific for the M phase. Cell cycle was arrested with hydroxyurea (‘HU'), temperature-sensitive mutations or nitrogen starvation (‘-N') at S, G1 or G2. Numbers in parenthesis indicate the number of nuclei examined. (**c**) An optical section of a typical anaphase nucleus (DAPI staining; blue) without a knob near the subtelomeric markers (Taz1-GFP in green and H3K9me3 in red). The lower panel shows DAPI staining in white marked with Taz1-GFP (green contours) and H3K9me3 foci (red contours) coexisting at a single chromosome end. The chromosomes are pulled in the direction of the arrows. The bar indicates 500 nm for **c**,**d**. (**d**) An optical section of a typical DAPI-stained nucleus containing two knobs, commonly found in HU-treated cells.

The genome of *S. pombe* has well-studied, discrete silent heterochromatic regions marked by histone H3 methylated at K9 (H3K9me). The subtelomeric and pericentromeric regions are the two largest heterochromatic regions (∼50 kb each) and are suitable for cytological analysis using 3DSIM because of their large size in *S. pombe*. Unexpectedly, known silent regions indicated by H3K9me3, as well as regions indicated by markers for the centromere (Cnp1-GFP) and the telomere (Taz1-GFP) had the lowest DNA concentration of the regions examined in this study (Fig. 2b,c). In particular, the Cnp1-GFP region is the innermost kinetochore region of the centromere (*cnt*) where foreign genes are known to be silenced^11^, but exhibited the lowest condensation level (Fig. 2b,c). Taz1 is the orthologue of TRF in mammals and recognizes telomere-associated sequences^12^. Although the length of the Taz1-binding sites could be too short to image with 3DSIM, the results showed that telomere-associated sequences were separate from any nearby condensed regions (Fig. 2c). H3K9me3 foci were found close to Cnp1- or Taz1-GFP foci, and often found in between the chromatin mass and the outside regions (Fig. 2c; see also Figs 3a, 4c and 5c). This was also the case when we used histone H3, instead of DAPI, as the chromatin marker (Fig. 2d, Supplementary Fig. 2a). The results for H3K9me2 were essentially identical to those for H3K9me3, although the antibody against H3K9me3 produced a more specific and intense signal than that against H3K9me2 (Supplementary Fig. 2b,c). In addition, the overlap with DAPI staining was similar for pericentromeric and subtelomeric H3K9me3 loci (Supplementary Fig. 2d,e). Silent regions marked by H3K9me3 (about 50 kb each) should be large enough to recognize with 3DSIM as we were able to visualize other chromatin regions of a similar size, that is, ‘knob' (see below). Decondensed silent chromatin was already predicted from reported micrococcal nuclease I (MNaseI) protection assays, where subtelomeric and pericentromeric heterochromatin regions had lower MNaseI protection than the rest of the genome^13^,^14^ (summarized in Supplementary Fig. 5; also see Fig. 7a). Also, origins of replication in pericentromeric regions and the silent mating-type locus are replicated early in S phase^15^,^16^. Furthermore, the *cnt* region of centromere is known to be modified by H3K4me3 (ref. 17). Collectively, the evidence indicates that silent regions in *S. pombe* are in fact less condensed than active regions.

### The most condensed chromatin flanks subtelomeres

A further unexpected finding was that a highly condensed chromatin body was adjacent to the subtelomeric DNA (Fig. 3a). It was close to, but did not overlap with, subtelomeric H3K9me3 foci near the Taz1-GFP foci (Fig. 3a). This body was not stained by most antibodies against common histone modifications (Fig. 3b and Supplementary Fig. 3a–d). Particularly active chromatin marks (RNAPII and H3K4me) were excluded from this region (Fig. 3b). We confirmed that this body was made of nucleosomes (Fig. 3c and Supplementary Fig. 3e). Antibody was not totally inaccessible in the chromatin body because it was stained well by anti-GFP antibody recognizing histone H2B-GFP (Supplementary Fig. 4). We named this type of chromatin body a ‘knob'.

The data presented above indicated that the knob region was close to subtelomeric H3K9me3 foci, and characterized by very low levels of H3K4me and RNAPII. We compiled the publicly available genome-wide chromatin immunoprecipitation (ChIP) and microarray data^17^,^18^,^19^,^20^ and found that such regions exist on the centromeric side of the subtelomeric regions at both ends of chromosomes 1 and 2, but not 3 (Supplementary Fig. 5). The centromeric side of subtelomeric regions was previously referred to as ‘ST-chromatin'^21^, where genes expressed in nitrogen starvation are enriched^22^ (Fig. 4a). We used *lacO* arrays inserted near the ST-chromatin and other regions in the genome, and detected the *lacO* arrays with lacI-GFP with 3DSIM (Fig. 4b,c and Supplementary Fig. 5). The *lacO* loci inserted close to the ST-chromatin regions (*sod2*, *AC1*, *AC26*, *CII-L*, *CII-R*) were found adjacent to the knob regions (Fig. 4b). However, both ends of chromosome III (*meu3*, *meu19*), centromeres (Cnp1-GFP), the silent mating-type locus (represented by *his2*) and a region of chromosome 1 (*AC24*), which shows a similar transcriptional profile to ST-chromatin^22^, were rarely found in close proximity (Fig. 4b). Furthermore, when the *lacO* array was inserted on the telomere side of the ST-chromatin (*sod2::lacO*) and on the centromere side of the ST-chromatin (*AC1::lacO*), those were found on the opposite sides of the knob relative to the telomeric H3K9me3 foci in the interphase nucleus (Fig. 4c), indicating that the knob is formed on the ST-chromatin. The same result was obtained in the meiotic ‘horsetail' nucleus, in which the chromosomes are bundled at the telomeres and aligned in the elongated horsetail-shape nucleus^23^ (Fig. 4d). Since ST-chromatin is located at both ends of chromosome 1 and 2, multiple knobs could be found in the nucleus. In fact, of the 402 wild-type nuclei examined, 196 nuclei (48.8%) contained one knob, and 19 nuclei (6.0%) contained two knobs. Since chromosome ends in *S. pombe* tend to cluster in interphase, smaller numbers of knobs are expected. These data showed that knobs were formed from ST-chromatin located at inner-subtelomeric regions of chromosome 1 and 2.

Since knob regions appeared to be clustered, we re-examined the previously reported genome-wide Hi-C results^24^. We found that telomeric regions with high physical proximity values indeed corresponded to ST-chromatin regions of chromosome 1 and 2, but not 3 (Supplementary Fig. 6). This tendency for clustering is consistent with our cytological observations and explains why only one or two knobs were observed in the nucleus.

### Knob condensation is dynamically regulated

Of the 402 wild-type nuclei examined, 182 nuclei (45.3%) contained no knobs in an asynchronous cell population. Time-lapse observation of living cells during the cell cycle showed that knobs were consistently observed in the G2 phase of the cell cycle, and disappeared at the mitotic phase (Fig. 5a). Knobs reformed some time after mitosis, and remained throughout the rest of interphase. To further examine how knob formation is controlled, we examined cells at each cell cycle stage. Cells at the M phase did not exhibit knobs, but almost all (92-100%) cells arrested at G1, S and G2 had knobs (Fig. 5b,c). These data indicate that knobs disappear in the M phase and are recreated in the subsequent interphase. It seems to take some time to form knobs during the G1 phase, but knob formation is complete if the cell cycle is arrested. For an unknown reason, a high proportion of cells arrested at the S phase had two knobs (Fig. 5b,d). Cells arrested at G1 with a temperature-sensitive cell cycle mutation *cdc10-129* showed knobs, but cells arrested at G1 with nitrogen starvation did not show distinct knobs (Fig. 5b). It is known that genes in ST-chromatin are expressed during nitrogen starvation^22^, suggesting that the gene expression may compromise knob formation. These data clearly show that the knobs are not ‘constitutive', and that their condensation is dynamically regulated by cellular conditions.

### Knob condensation is controlled by histone epigenetic marks

**Figure 6:**
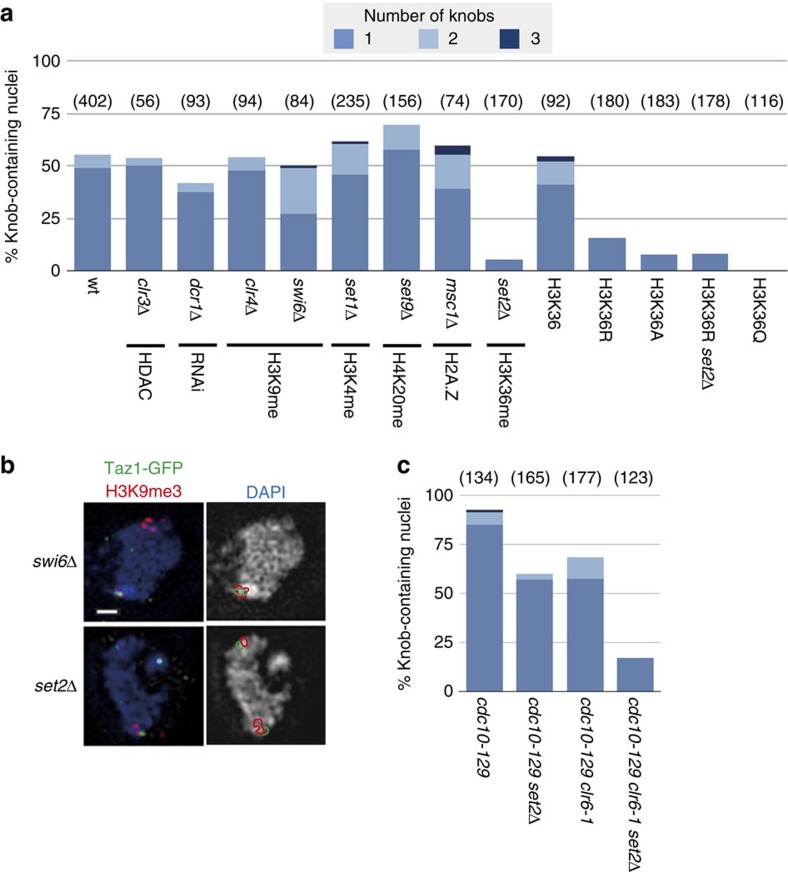
Knob condensation is independent of gene silencing. (**a**) Frequency of knob-containing nuclei in various mutants grown under standard conditions. The affected pathways are indicated below the genotypes. (**b**) Knobs in *swi6*Δ and *set2*Δ mutant cells. Taz1-GFP (green) and H3K9me3 (red) images are merged with the DAPI images (blue) in the left panels. The right panels show the DAPI images (white) superimposed with Taz1-GFP and H3K9me3 regions (green and red contours, respectively). A knob is clearly visible in *swi6*Δ but not in *set2*Δ. Scale bar, 500 nm. (**c**) Frequency of knob-containing nuclei in various mutants grown in YES at 36 °C. The temperature-sensitive allele of *cdc10* was used to arrest the cell cycle at G1. Numbers in parenthesis in **a** and **c** indicate the number of nuclei examined.

We examined if gene silencing factors play a role in the DNA condensation in knob regions. Genes *clr3*, *dcr1*, *clr4* and *swi6* are all required for gene silencing^25^,^26^. Mutants in which these genes were disrupted, however, showed normal knob formation (Fig. 6a,b). These data demonstrated that the most condensed DNA regions in *S. pombe* chromatin were unrelated to canonical gene silencing.

Since ST-chromatin was characterized by the absence of modifications of histone H3 tails and histone variant H2A.Z^21^ (Supplementary Fig. 5), we examined knob formation in mutants defective in epigenetic marks. H3K4 (*set1*)^27^ and H4K20 (*set9*)^28^ methyltransferase mutants showed no apparent defects in knob formation (Fig. 6a). Deposition of H2A.Z at the ST-chromatin region is controlled by *msc1* (ref. 21), but deletion of it did not impair knob formation either (Fig. 6a). In contrast, a dramatic effect was observed with deletion of the sole methyltransferase for H3K36 (*set2*)^29^, that is, knobs were rarely found in the *set2*Δ mutant (Fig. 6a,b). To examine if Set2 acts through the methylation at K36 of histone H3, we constructed strains with a single histone H3 gene with an amino-acid substitution at lysine 36 to arginine or alanine (hereafter referred to as H3K36R and H3K36A). Knob formation was also reduced in these strains (Fig. 6a). The frequency of knob formation was similar in a H3K36R and *set2*Δ double mutant strain (Fig. 6a). Since H3K36R and H3K36A also lost acetylation at this lysine residue, we examined whether acetylation at K36 is important for knob formation by substituting a glutamine (Q) residue at K36, to mimic acetylation. To our surprise, the acetylation mimic mutant, H3K36Q, showed a total loss of knob formation (Fig. 6a), suggesting that acetylation at K36 is sufficient for knob decondensation. Collectively, these data indicate that the epigenetic marks at H3K36, but none of the other histone modifications examined, play a critical role in knob formation.

The above data suggest that deacetylation of H3K36 is important for maintaining knob condensation. The histone deacetylase Rpd3 is known to interact with methylated H3K36 in *Saccharomyces cerevisiae*^30^,^31^. Because the gene encoding its orthologue, Clr6, in *S. pombe* is essential for vegetative growth, we examined the phenotype of a strain harbouring a temperature-sensitive allele, *clr6-1*, at a restrictive temperature. To produce comparable conditions, even under possible cell cycle arrest due to *clr6-1*, the cell cycle mutation *cdc10-129* was included in all strains examined. At the restrictive temperature, almost all nuclei in the control strain contained knobs, due to cell cycle arrest (Fig. 6c, see also Fig. 5). The *set2Δ* mutant showed modest knob formation under these conditions (Fig. 6c), suggesting that the condensation machinery itself remains intact in this mutant. Knob formation in the *clr6-1* mutant was reduced to a level similar to that of *set2*Δ (Fig. 6c). Surprisingly, a *set2Δ* and *clr6-1* double mutant showed a very low frequency of knob formation (Fig. 6c). Thus, in addition to methylation at H3K36 by Set2, deacetylation by Clr6 is also important for knob formation.

### Knob condensation affects subtelomeric gene silencing

**Figure 7:**
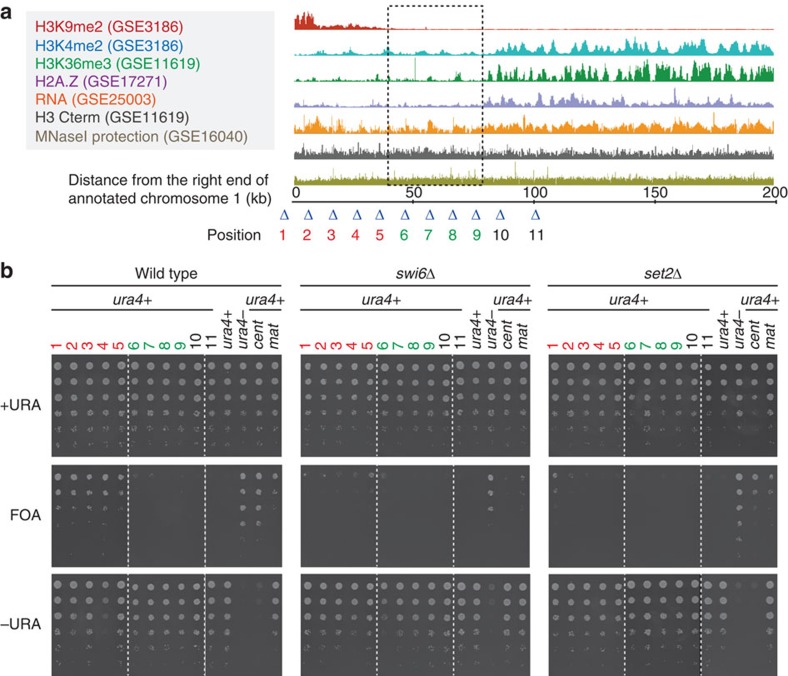
Foreign genes inserted into the knob region are not silenced. (**a**) A compiled summary of publicly available ChIP, RNA level or MNaseI protection data for the 200-kb region extending from the end of annotation of the right arm of chromosome 1. The colour code for the ChIP, RNA level and MNaseI data is shown on the left with their corresponding accession numbers. The ST-chromatin is indicated with a dotted box. Triangles indicate *ura4*+ gene insertion sites in the subtelomere (1-5, red), ST-chromatin (6-9, green) and euchromatin (10-11, black) regions. (**b**) Fivefold serial dilution of the indicated strains grown on the indicated plates. Strains labelled with ‘*cent*' have *ura4*^+^ inserted at the dg repeat of the pericentromere while ‘*mat*' has *ura4*^+^ inserted at the silent mating-type locus.

Since knobs were found to be highly condensed, we considered that genes might be silenced in the knobs. Thus, we examined gene silencing at the right subtelomeric region of chromosome 1 by *ura4*^+^ gene insertion (Fig. 7a). If *ura4*^+^ is repressed, cells are able to grow in the presence of toxic analogue 5-fluoroorotic acid, but unable to grow in the absence of uracil. In the wild-type background, subtelomeric silencing was observed only in the region where H3K9 was methylated (Fig. 7b, ‘FOA' positions 1–5), but no gene silencing was observed in ST-chromatin (Fig. 7b, ‘FOA' positions 6–9). These data indicate that knob formation had little effect on the foreign gene repression.

In *swi6*Δ, although most gene silencing disappeared at the subtelomeric region, slight gene silencing remained at the boundary between ST-chromatin and silent chromatin (Fig. 7b, positions 5–6).

Surprisingly, in *set2*Δ, most subtelomeric silencing disappeared, particularly in the regions close to the ST-chromatin (Fig. 7b). Gene silencing at pericentromeric regions and silent mating-type locus was also slightly weakened in *set2*Δ (Fig. 7b), suggesting that *set2* exerts weak effects on gene silencing in general but has a stronger effect on subtelomeric regions. This is the first demonstration that *set2* is required for gene silencing.

### Gene activation is associated with knob decondensation

**Figure 8:**
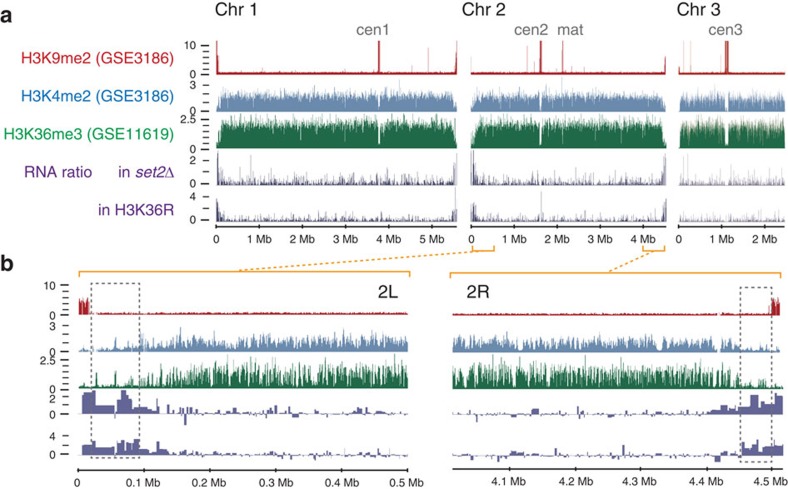
Genes in subtelomere and ST-chromatin regions are derepressed in the absence of H3K36 methylation. The log2 ratio of RNA levels in *set2*Δ or H3K36R cells to their control strains are plotted along with histone modification levels (accession numbers are given in parentheses). (**a**) All chromosomes are shown. The labels ‘cen1', ‘cen2', ‘cen3' and ‘mat' indicate the positions of the centromeres of chromosomes 1, 2 and 3 and the silent mating-type locus, respectively. The 500-kb regions extending from each end of chromosome 2 are magnified and presented in **b**. The dotted boxes show the position of the ST-chromatin.

To examine the relationship between chromatin condensation at knob regions and gene expression in more detail, global gene expression levels in *set2*Δ and H3K36R cells were examined. Derepression in *set2*Δ and H3K36R cells was observed specifically at the subtelomere of chromosomes 1 and 2 but not 3 (Fig. 8a). Here again, derepression was more specific to subtelomeric regions and little elevation was found in pericentromeric regions and the silent mating-type locus (Fig. 8a). Higher-resolution inspection of the subtelomeric region (Fig. 8b) clearly showed that derepression was not only found in subtelomeric silent regions, but extended to the ST-chromatin where knobs were formed. Taken together, these results demonstrate that decondensation of knobs is associated with gene activation.

Since the methylation status of H3K36 affects gene expression, it is possible that a key regulatory gene was upregulated or downregulated by loss of H3K36me, which in turn derepressed gene expression at subtelomeres and decondensed knobs. Thus, we examined the genes that showed greater than twofold elevation or repression in three strains—*set2*Δ, H3K36R and H3K36A (Supplementary Fig. 7a). No genes were downregulated more than twofold in all of the three strains. Of the 142 genes found to be upregulated in all three strains, 89 were protein-coding genes (63%), 44 were non-coding RNA genes (31%) and 9 were pseudogenes (6%); the proportion of protein-coding genes was significantly lower and the fraction of pseudogenes was significantly higher than the expected ratio, based on random selection using a DNA microarray probe (*χ*^2^-test, *P*<0.001; see Supplementary Fig. 7b). Gene ontology (GO) analysis of the 89 protein-coding genes showed that a significantly large proportion of the genes were associated with an unknown biological process (31%) or molecular function (26%) (*χ*^2^-test, *P*<0.0002, Supplementary Fig. 7c). Of the genes that could be assigned to a known GO category, a significantly large proportion was involved in cell adhesion, transmembrane transport and metabolic processes (*χ*^2^-test, *P*<0.0002, Supplementary Table 1). We assumed that factors which may affect transcription of knob regions are localized in the nucleus. We identified 31 genes for which the ‘nucleus' is annotated as the ‘cellular component' (Supplementary Table 2). Of these 31 genes, seven were mapped to subtelomeric silent regions. If any of these genes are responsible for knob formation, knobs are expected to disappear when their transcription is elevated in gene silencing mutants (for example, *swi6*Δ); however, these mutants showed normal knob formation (see Fig. 6), indicating that these seven genes are not responsible for knob formation. Sixteen genes were mapped to knob regions. Two of them encode predicted transcription factors, but we cannot eliminate the possibility that they regulate their own transcription inside the knob. Of the eight remaining genes, only *cbf12* encodes a known transcription factor. We examined the publicly available genome-wide transcription data for cells overproducing Cbf12 (ref. 32; GSE41730), and found that main chromosome bodies showed a higher degree of upregulation than knob regions (Supplementary Fig. 8). These data thus suggest that the loss of methylation at H3K36, rather than secondary effects of other transcriptional regulators, led to the activation of cryptic promoters in the knob regions.

## Discussion

Using super-resolution microscopy, we are now able to examine the spatial organization of the genome within the small nucleus of *S. pombe*. Our results on *S. pombe* chromatin challenge the classical view of interphase chromatin structures. In particular, we found that the silent chromatin was highly decondensed; nevertheless, its transcription was repressed in that region. The facts that the silent chromatin in *S. pombe* exhibits high MNaseI sensitivity^13^,^14^ and early replication in the S phase^15^,^16^ also support our cytological observations. Therefore, we conclude that the silent chromatin is indeed decondensed in *S. pombe*. In contrast, foreign genes inserted into the highly condensed knob region were not silenced. These results suggest that chromatin condensation alone does not repress gene expression.

We demonstrated that in *S. pombe*, the unusual condensed chromatin knobs formed at the regions adjacent to, rather than directly on, the silent subtelomeric regions. The knob regions are known to function as ‘neocentromeres' when an authentic centromere is artificially removed^33^. Interestingly, the classical ‘knobs' identified in maize also function as neocentromeres^34^. In *S. pombe*, the central regions of the centromere and the knob regions share an uncommon characteristic, namely they have very low levels of methylated histones^21^ and have neighbouring silent H3K9me2/3 regions^33^. Despite these resemblances, in interphase, knobs were highly condensed but centromeres were highly decondensed. Examination of additional ChIP-seq data may reveal as-yet-unknown factors that characterize knobs.

We further demonstrated that epigenetic marks, namely methylation and acetylation at H3K36, play a crucial role in chromatin structure and gene repression at knob regions in *S. pombe*. Set2 is the sole methyltransferase for H3K36 in *S. pombe*, but no obvious phenotype is observed in vegetative growth, except for slow growth in synthetic media^29^. The *set2*Δ and H3K36R strains show a similar phenotype and gene expression—derepression of gene expression in *set2*Δ or H3K36R was mostly limited to the ends of chromosomes 1 and 2, and no other regions of the genome were greatly altered. Detailed inspection of gene expression in these strains revealed that the majority of upregulated genes were non-coding RNA, pseudogenes and genes without known GO. These data are concordant with the conserved function of methylated H3K36, repression of cryptic promoters and inhibition of transcription elongation^30^,^31^,^35^. Thus, a major role of H3K36 methylation is regulation of the structure and function of ST-chromatin and the subtelomeric silent chromatin in *S. pombe*. Epigenetic marks at H3K36 may act as a boundary to separate subtelomeric chromatin from classical ‘euchromatin'.

## Methods

### *S. pombe* strains and growth conditions

The *S. pombe* stains used in this study are summarized in Supplementary Table 3. Cells were cultured using standard methods. For microscopy, cells were cultured in liquid minimum medium with supplements (EMM2 5S) at 26 °C with shaking to the early logarithmic phase. Phenotypes of temperature-sensitive mutants were observed after 3 h of culture at 36 °C in EMM2 5S, except the experiments shown in Fig. 6c, where cells were grown in YES medium. To arrest at the S phase, 15 mM HU was added and the cells were cultured for 24 h at 26 °C. For the serial dilution experiments, synthetic defined medium (0.67% Bacto yeast nitrogen base without amino acids and 2% glucose) was used with 225 mg l^−1^ histidine, leucine, lysine, with or without 225 mg l^−1^ uracil, and with or without 1 g l^−1^ 5-fluoroorotic acid (Wako, Osaka, Japan).

### Construction of transgenic *S. pombe* strains

Construction of transgenic *S. pombe* followed standard methods. To observe live cells with 3DSIM, we used special GFP fusion histone constructs (Supplementary Fig. 1a). For the GFP fusion of H2B, *hta1*, *htb1*, their native promoter (the whole region between *hta1* and *htb1*) and their 3′ region (636 bp downstream of *hta1* and 866 bp downstream of *htb1*) were cloned into a plasmid, and inserted next to the *lys1* locus. For the GFP fusion of H3, *hhf2*, *hht2*, their native promoter (the whole region between *hhf2* and *hht2*) and 3′ region (556 bp downstream of *hhf2* and 503 bp downstream of *hht2*) were inserted next to the *lys1* locus. When GFP was fused to the native H3 gene, the terminator from *adh1* was used for the fusion gene. A ten amino-acid linker peptide (NH_2_-AAGGGSGGGS) was used for all of the GFP fusion proteins with the exception of GFP-H3, which did not use a linker.

Chromosome loci were visualized by the insertion of *lacO* repeat arrays and expression of a LacI-GFP fusion under the control of the *dis1* gene promoter^36^,^37^,^38^. A two-step integration method was used to ensure high-efficiency integration of *lacO* arrays into a target position^39^. In brief, the first PCR step amplified ∼300 bps of DNA sequence downstream and upstream of the target *lacO* insertion position using genomic DNA as a template. Next, the second PCR step amplified a partial *ura4* gene and a kanamycin gene cassette in a plasmid pCT33-6 using the PCR product from the first step. The amplified partial *ura4*-containing DNA fragment was integrated at the target locus using the *kan*^*r*^ gene as a selection marker for G418. Subsequently, a plasmid pCT31 containing the *lacO* repeats was integrated into the locus using the *ura4* sequence as a target. Integration was confirmed by colony PCR and DNA sequencing.

### Sample preparation for imaging

Antibodies used in this study are listed in Supplementary Table 4.

Cells in the early logarithmic phase were pelleted by gentle centrifugation, and chemically fixed by resuspending in a buffer containing 4% formaldehyde (Polysciences Inc., Warrington, USA), 80 mM HEPES-K, 35 mM HEPES-Na, 2 mM EDTA, 0.5 mM EGTA, 0.5 mM spermidine, 0.2 mM spermine and 15 mM 2-mercaptethanol, pH 7.0. This fixation buffer was designed by combining two buffers, (i) ‘buffer A', known to maintain polytene chromosome structure in the fly^40^ and (ii) HEPES buffer that maintains RNAPII foci by counteracting the fall in pH, which occurs during the crosslinking process induced by aldehyde^41^. Cells were fixed for 10 min at room temperature, then washed with PEMS (100 mM PIPES, 1 mM EGTA, 1 mM MgSO_4_, 1.2 M sorbitol pH 6.9) three times. Each washing step used 2–5 min of rotation. We included 1.2 M sorbitol in all steps of sample preparation to maintain the constant osmotic pressure in live cells except the fixation step where 4% formaldehyde alone corresponds to 1.33 M. Then, the cell walls were digested with 0.6 mg ml^−1^ zymolyase 100T (Seikagaku Biobusiness, Tokyo, Japan) in PEMS at 36 °C for 5 min (for DAPI staining) or 30 min (for immunostaining). We used relatively short digestion times since cells and the nuclei of cells whose walls had been completely digested were easily flattened when they were mounted for imaging. Next, cells were treated with 0.1% Triton X-100 in PEMS for 5 min, and washed three times thereafter with PEMS. For DAPI staining, cells were incubated with 0.2 μg ml^−1^ DAPI for 10 min, and mounted with *n*PG-Glycerol (100% glycerol for absorptionmetric-analysis (Wako, Osaka, Japan) with 4% *n*-propyl gallate, pH 7.0). For immunostaining, cells were incubated with 1% bovine serum albumin, 0.1% NaN_3_, 100 mM L-lysine monohydrochloride in PEMS (PEMSBAL) for 30 min, and the solution was replaced with antibody in PEMSBAL and incubated for 1 h. Cells were washed with PEMSBAL three times and then the secondary antibody was added. Afterwards, the cells were washed, counterstained with DAPI and suspended in mounting medium (*n*PG-Glycerol, see above). A drop of 1.35 μl of cell suspension in *n*PG-Glycerol was put on a clean 18 × 18 mm coverslip (No. 1S, Matsunami, Osaka, Japan). The coverslip was then put on a glass slide letting the solution spread over the area of the coverslip. Owing to the small volume of the surrounding medium, cells were caught between the two glass surfaces and Brownian motion ceased. Then, the coverslip was sealed with rubber cement, and the cells imaged within 24 h.

### Image acquisition

We used a DeltaVision|OMX microscope version 3 (GE Healthcare, Buckinghamshire, UK) for 3DSIM imaging with a × 100 UPlanSApo NA1.40 oil immersion objective lens (Olympus, Tokyo, Japan). For image data that were subsequently used for quantitative measurements (overlap), we used the same immersion oil with a refractive index (RI) of 1.514 and used only blue and green channels, with the exception of some experiments summarized in Supplementary Figs 2 and 4b, where immersion oil with a RI of 1.518, and red and green (Supplementary Fig. 2a, H2B-GFP and H3-GFP) or blue (Supplementary Figs 2b–e and 4b) channels were used. For live 3DSIM, cells in EMM2 5S attached to glass-bottom dishes (MatTek Corporation, Ashland, USA) coated with soybean lectin (Sigma-Aldrich, St. Louis, USA), were imaged with immersion oils with a RI of 1.520–1.522 (Supplementary Fig. 1b–e). The temperature around the sample stage was ∼27±1 °C. For deconvolution microscopy, a DeltaVision Elite microscope (GE Healthcare) with a × 60 PlanApo NA 1.4 oil SC objective lens (Olympus) was used.

### Image processing and presentation

Reconstruction of 3DSIM was performed by softWoRx (GE Healthcare) with Wiener filter constants between 0.002 and 0.004. For live 3DSIM, we used Wiener filter constants of 0.012. We used our own optical transfer functions for each colour averaged over many optical transfer functions. For constrained iterative deconvolution^42^, Priism suite (http://msg.ucsf.edu/IVE/) was used with a Wiener filter enhancement of 0.9 and 15 iterations. Pictures for image presentation were prepared with Priism suite without changing gamma.

### Adaptive image registration

Since image registration of multicolour images was not accurate when performed by the software of the microscope supplier, we made our own registration software. The programme used correlation to find the best parameters for translation in *x*, *y* and *z* axes, as well as *z* axis rotation and magnification in *x*, *y* and *z* axes. Since the OMX microscope uses different EMCCD (and the optical components) for each channel, modifications in magnification for two axes were required to correct for the astigmatism of the focusing lens of each channel. Although we prepared the registration parameters using multicolour fluorescent beads (Invitrogen, Carlsbad, USA), values for *x*, *y* and *z* translation were adaptively corrected for the biological images every time we took images. This is because chromatic aberration in ideal bead samples was different from that of biological samples. Furthermore, even among slides of biological samples, the distances between the coverslip and the sample were always different, and the thickness of the coverslip at each location could be slightly different as well. For these reasons, we often observed different registration parameters for different *z* stacks, particularly in *z* translation. To correct for this sample-dependent chromatic aberration, we imaged DAPI signal in all required channels (Supplementary Fig. 9). Since the emission wavelength of DAPI extends over 600 nm, it is possible to reconstruct 3DSIM images using only DAPI signals excited with a 405-nm laser. The registration parameters were created with these DAPI images using the beads alignment as the initial guess. These DAPI images were taken after acquiring multicolour images to prevent bleaching of the samples. A detailed description of channel alignment will be published elsewhere.

### Algorithm for the co-localization analysis

Co-localization analysis was done on the individual nuclei in 3DSIM images. To segment nuclei, binary images were first made by thresholding.

For the thresholding, we used automatic iterative thresholding^43^, which adaptively finds the threshold values from the given image. We introduced a single parameter *d* for flexible thresholding.


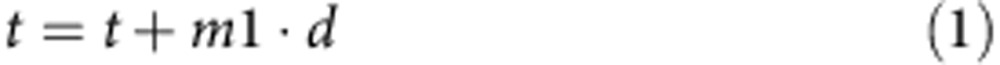


where *t* is the threshold calculated by automatic thresholding and *m*1 is the mean of pixels above threshold used in the calculation of *t*. Keeping the parameter *d* at similar values, we were able to obtain thresholds that were visually constant. The threshold was always visually inspected and modified when necessary.

Next, individual DAPI-stained nuclei were found using our programme using functions from the ‘ndimage' module of the Scipy package (http://www.scipy.org). Using DAPI as the reference, signals in other channels within a distance of 0.2 μm from the object in the blue channel were grouped as a single object (nucleus).

To measure the overlap of the fluorescence signals with DAPI, we used modification of *k* and *M* values proposed by Manders *et al*.^44^. The detailed description and validation will be published elsewhere. Here we briefly explain its theoretical background.

The first step of our method prepares cropped images with or without thresholding (see above) and then normalized. The image thresholding and normalization (if the original image is *G*, then the resulting image is called *G*_*t,*norm_) was done by the following formula:


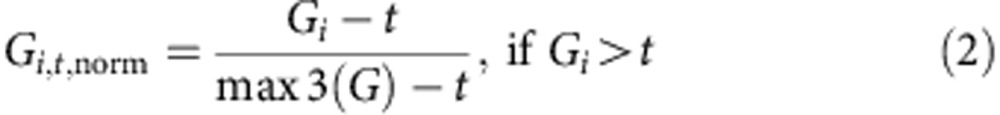



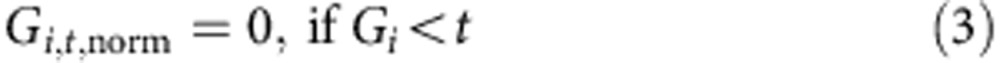


where *t* is the threshold and max3 is a function that averages the top three pixels in the intensity of the object. For normalization, usage of three pixels from the top buffers the possible noise in the image. Three pixels were enough for this purpose since the reconstruction process of 3DSIM already removed high-frequency noise (for example, salt-and-pepper noise). For a technical reason, the images were padded to 250 times by pixel volume with zero values to precisely calculate the ratio of variance. The overlap (*n*) of the immunofluorescence signal (*G*) with the DAPI signal (*B*) was then calculated as follows:


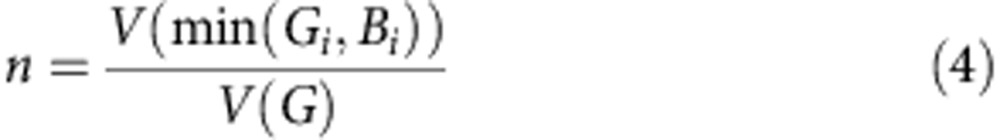


where *V* is a function to obtain the variance of the whole image.

### Counting knobs

Conspicuously condensed, DAPI-stained bodies were counted as knobs by visually inspecting each optical section of 3DSIM images.

### Processing of ChIP and microarray data

The processed data were downloaded from the ArrayExpress (https://www.ebi.ac.uk/arrayexpress/) or NCBI GEO (www.ncbi.nlm.nih.gov/geo/) databases. We constructed simple programmes to convert the processed data into the sgr or bedGraph formats. Data are presented using the Integrated Genome Browser^45^. The GO assignments were downloaded from PomBase (http://www.pombase.org) and the GO terms were downloaded from the Gene Ontology Consortium (http://geneontology.org). The *χ*^2^-test was used for analysis, with an expectation of a random distribution using whole-genome data or the set of all probes used for the microarrays.

### Processing of Hi-C data

Distance-normalized physical proximity data for Hi-C were downloaded from the website at http://www.wistar.org/research_facilities/noma/pubdata.htm. The data were given the filename ‘original,' but the content was ‘distance-normalized,' according to the paper^24^. We wrote a simple programme to convert the data into images using ‘section 1' as the *y* coordinates and ‘section 2' as the *x* coordinates.

### RNA preparation for DNA microarray

Cells were grown in YES liquid medium at 30 °C and collected with filtration when they reached a density of 5 × 10^6^ cells ml^−1^. The cell pellet was frozen with liquid nitrogen, and resuspended in 500 μl of ice-cold TES (10 mM Tris pH7.5, 10 mM EDTA pH8, 0.5% SDS). Then, 500 μl of acidic phenol–chloroform was added to the cell suspension and mixed immediately. The sample was incubated at 65 °C for 1 h, and then placed on ice for 1 min. The sample was centrifuged for 15 min at 15,100*g* at 4 °C. The water phase of the sample was recovered, and extracted with acidic phenol–chloroform and then washed with chloroform using phase-lock tubes (MaXtractTM HighDensity 2 ml Qiagen, Valencia, USA). Finally, total RNA from 500 μl of the water phase was precipitated with ethanol.

### DNA microarray experiments

Cyanine-3 (Cy3)-labelled cRNA was prepared from 200 ng of total RNA using the LowInput QuickAmp Labeling Kit one-colour including Cy3-CTP (Agilent Technologies, Santa Clara, USA) according to the manufacturer's instructions, followed by purification using RNeasy mini column (Qiagen). The Gene Expression Hybridization Kit (Agilent Technologies) was used for hybridization according to the manufacturer's instructions except that 37.5 ng of Cy3-labelled cRNA was used for each hybridization. After hybridization, microarray slides were washed for 1 min at room temperature with GE Wash Buffer 1 (Agilent Technologies) and for 1 min at 37 °C with GE Wash buffer 2 (Agilent Technologies), then dried immediately. The microarray slides were then immediately scanned with the Agilent DNA Microarray Scanner (G2505C) with the following parameters—one-colour scan setting for 8 × 60 k array slides, scan area 61 × 21.6 mm, scan resolution 3 μm, dye channel set to Green and green PMT set to 100%. The scanned images were analysed with Feature Extraction Software (ver. 10.7.3.1, Agilent Technologies) using default parameters (protocol GE1-107_Sep09 and Grid: 038274_D_F_20120123) to obtain background subtracted and spatially detrended intensities. The 6,767 probes (including 6,711 *S. pombe* sequences and 56 control probes) were spotted multiply to fill 62,976 spots on each array. A median of ‘gProcessedsignals' of the spots for each probe was used to represent the intensity value of the probe. The median was normalized by the 75 percentile method (the value at 75% was set to 2,500). The probe signal is considered to be more than the detection limit when the average of ‘gIsWellAboveBG' of the spots for each probe is higher than 0.55. The measured values for these spots were assigned to the 6657 *S. pombe* genes.The intensity values of probes for a duplicated gene were divided by the copy number of the gene. Similarly, the values of probes that were cross-hybridized with paralogues were corrected using our custom correction probes. The list of correction probes and the detailed correction procedure with examples were given in the supplementary text files for the platform information deposited in GEO (GPL18374). In brief, sequences of probes for two paralogous genes were designed at exactly the same position in their coding sequences. In addition, correction probes were designed at the same positions each with a mismatch using either of two other bases. Assuming all mismatches weaken the hybridization efficiency to the same extent, then the intensity values of the target probes free from cross-hybridization can be found by solving linear equations using intensity values of paralogous and correction probes. For strains deleted for the *hhf1*^*+*^ and *hhf3*^*+*^ genes, however, we assigned surrogate values to *hhf1* and *hhf3* because the signals are obviously produced by cross-hybridization with mRNA of *hhf2*. The data from two biological replicates were combined by taking a geometric mean, and then divided by the values from control stains. As control stains, 968 was used for *set2*Δ (YAM073), and HA1050-5B was used for H3K36R (YAM233) and H3K36A (YAM232).

## Additional information

**Accession codes**: The sequences of probes and original data from the microarray experiments were deposited into the Gene Expression Omnibus database at National Center for Biotechnology Information under the accession code GSE63826.

**How to cite this article:** Matsuda, A. *et al*. Highly condensed chromatins are formed adjacent to subtelomeric and decondensed silent chromatin in fission yeast. *Nat. Commun*. 6:7753 doi: 10.1038/ncomms8753 (2015).

## Supplementary Material

Supplementary Information Supplementary Figures 1-9, Supplementary Tables 1-4 and Supplementary References
