# Supplementary material for: Highly condensed chromatins are formed adjacent to subtelomeric and decondensed silent chromatin in fission yeast
Source: Nat Commun. 2015 Jul 24;6:7753. doi: 10.1038/ncomms8753 (PMC4525289; doi:10.1038/ncomms8753)
Supplement: Supplementary Information — Supplementary Figures 1-9, Supplementary Tables 1-4 and Supplementary References [file ncomms8753-s1.pdf]

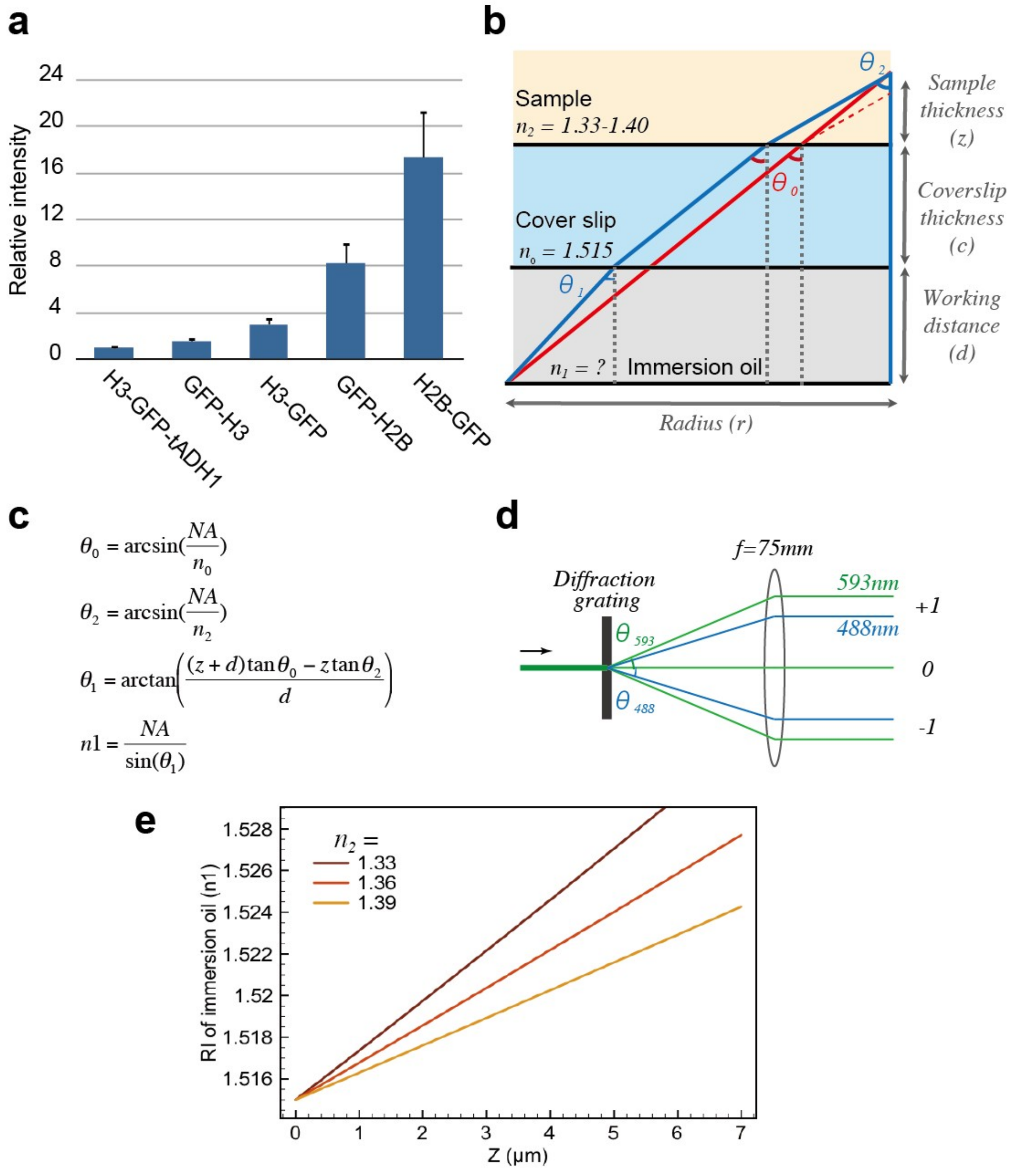

Supplementary Figure 1 Experimental and theoretical backgrounds for 3DSIM of live cells

(a) Fluorescence of GFP-fused histones used in this study. GFP-fused histone genes with their native 3’ UTR exhibited higher fluorescence intensity in the nucleus than widely used H3GFP gene with a foreign 3’ UTR (*adh1*).

(b) Geometrical representation of the left half of the three beams (indicated by blue or red lines) meeting at the focal plane (top). The objective lens is at the bottom. Excitation light passes through the immersion oil, cover slip and sample and each respective refractive index is indicated on the left. With the ideal refractive index, light beams are supposed to go straight (red line). Therefore, the focusing plane (where three beams meet each other) corresponds exactly to the imaging plane. However, in non-ideal mounting media such as water (n=1.333) or cytoplasm (n=1.36-1.40), light beams bend and do not meet at the imaging plane (dotted red line). They meet at the correct imaging plane if light beams are bent to the other direction once with the immersion oil (*n1*) with higher refractive index (blue line).

(c) Simplified math to get compromised *n1* from the known values. Terms are indicated in (b). NA=numerical aperture.

(d) NA of structured illumination in (c) is determined both by the relay lens (we used a 75 mm focal length) immediately after the diffraction grating, and wavelength of the excitation laser.

(e) Calculated refractive indices with a 488 nm laser and immersion oil *n1* that depends on sample thickness (*z*) and refractive index of the mounting medium (*n2*). For nuclei of living *S. pombe* (*z* = 2-3 μm) at 27 ˚C, we obtained the best result with immersion oil with refractive index (RI) 1.520.

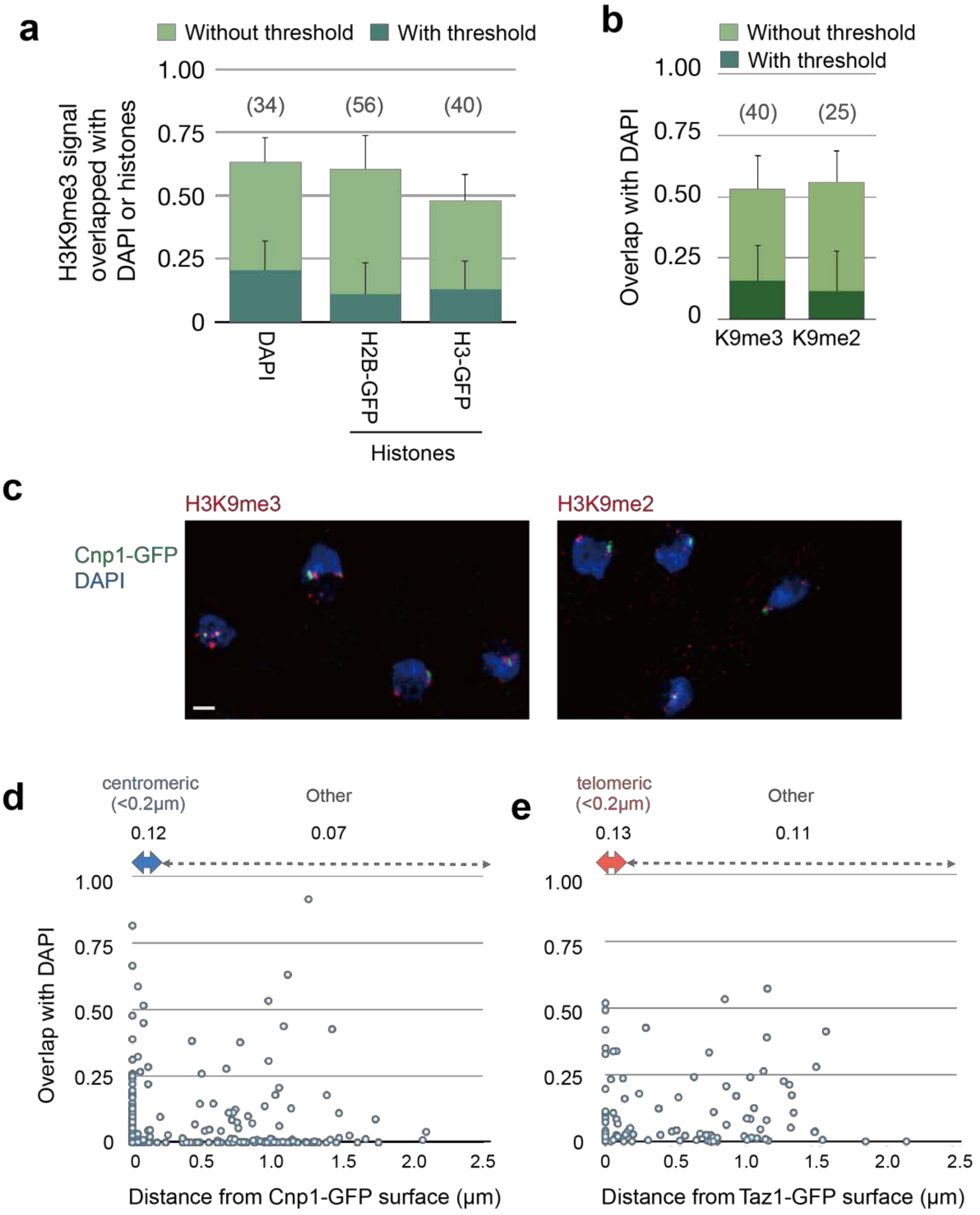

Supplementary Figure 2. DNA concentration of pericentromeric and subtelomeric silent loci

(a) Overlap of H3K9me3 with DAPI or histone-GFP. Bar shows standard deviation among nuclei. Numbers in parenthesis indicate the number of nuclei examined.

(b) Overlap of H3K9me3 or H3K9me2 with DAPI. Bar shows standard deviation among nuclei.

(c) Projected images of DAPI-stained nuclei immunostained with the indicated antibodies. Bar corresponds to 1 μm.

(d-e) Measurements of overlap of individual H3K9me3 foci with the DAPI signal plotted against the distance from the centromere (b) or telomere (c). The overlap was calculated from images with a threshold (see "Algorithm for the colocalization analysis" in Methods). Average values of overlap are indicated above each plot. A value of 1 indicates complete overlap.

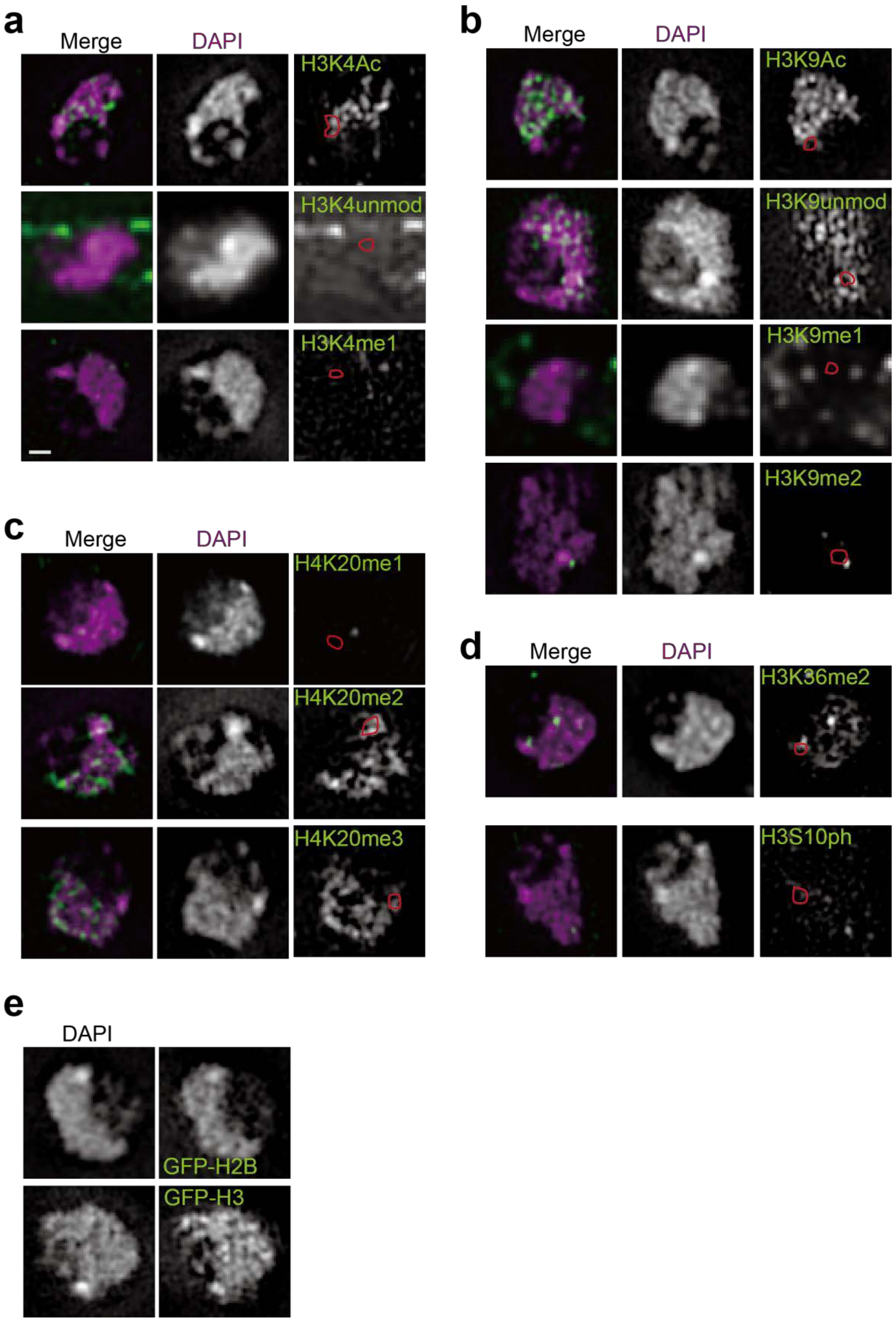

Supplementary Figure 3. Histone modifications in Knob regions

(a-f) Single optical sections taken with 3DSIM of immunostained nuclei using antibodies recognizing the indicated epitopes. The bar in (a) corresponds to 500 nm and the same scale was used for the images in (b-f). In the right panels of parts (a-d), the knob regions are indicated by red contours superimposed on histone modification images. See also Figure 3 for the rest of the modifications.

(a) Modifications at H3K4. Due to very weak staining, deconvolution was used for H3K4unmod (unmodified) instead of 3DSIM.

(b) Modifications at H3K9. Due to very weak staining, deconvolution was used for H3K9me1 instead of 3DSIM.

(c) Methylation at H4K20.

(d) Other histone modifications.

(e) Core histones N-terminally fused with GFP were present in knobs.

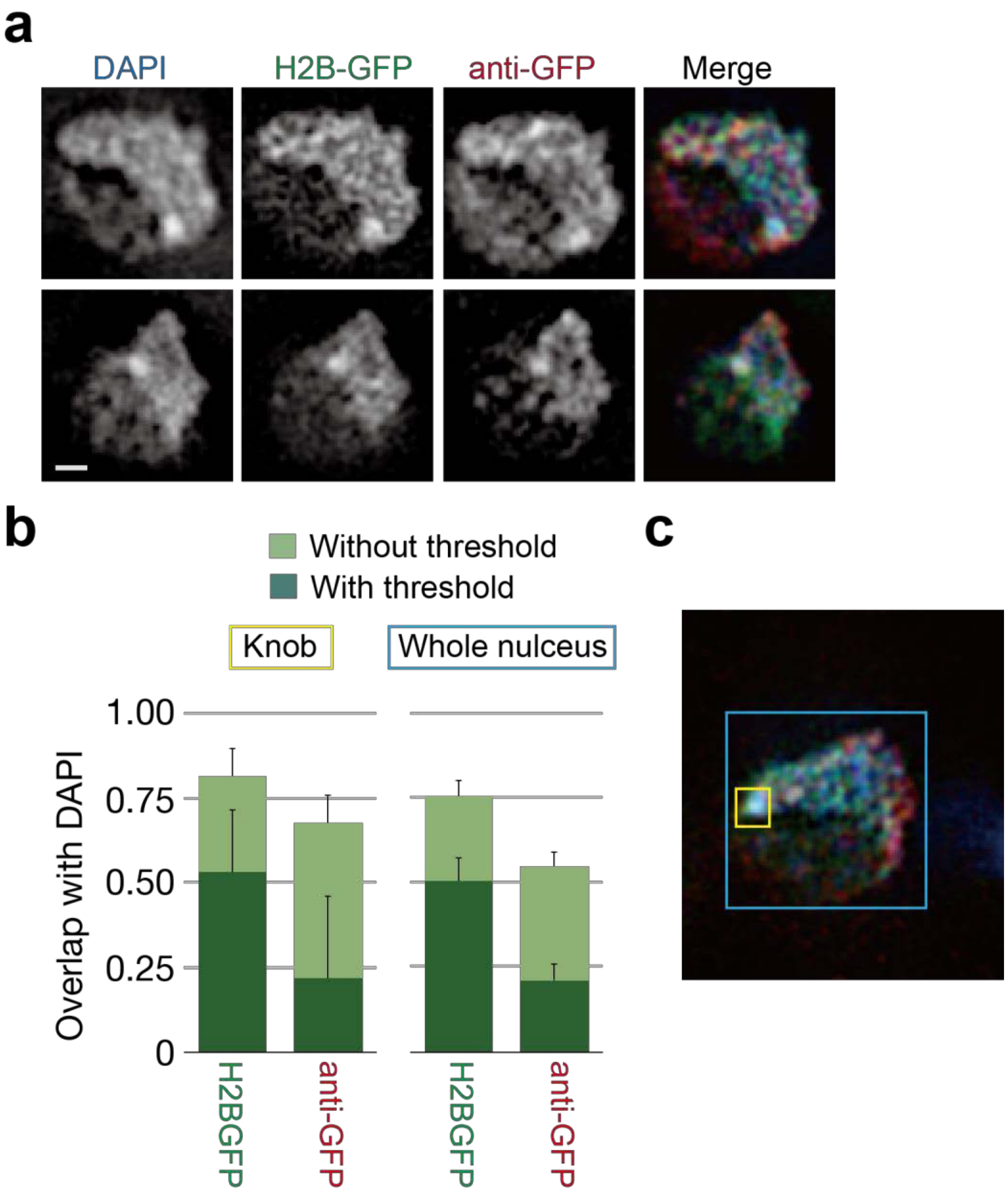

Supplementary Figure 4. Antibody accessibility of knobs

(a) Images of single optical sections captured through 3DSIM of H2B-GFP fluorescence (which inevitably appears noisy due to bleaching) combined with immunostaining, with red fluorescence indicated as "anti-GFP." Two representative nuclei are shown. The bar indicates 500 nm. (b) Overlap of GFP fluorescence with immunostaining for H2B-GFP

with DAPI. Regions (XY) containing only knobs were isolated and measured (*n*=7). Because immunostaining does not always stain all available epitopes, whole nuclei were also isolated and measured for comparison. (c) A representative nucleus showing selected knob (yellow) and whole nucleus (blue) regions.

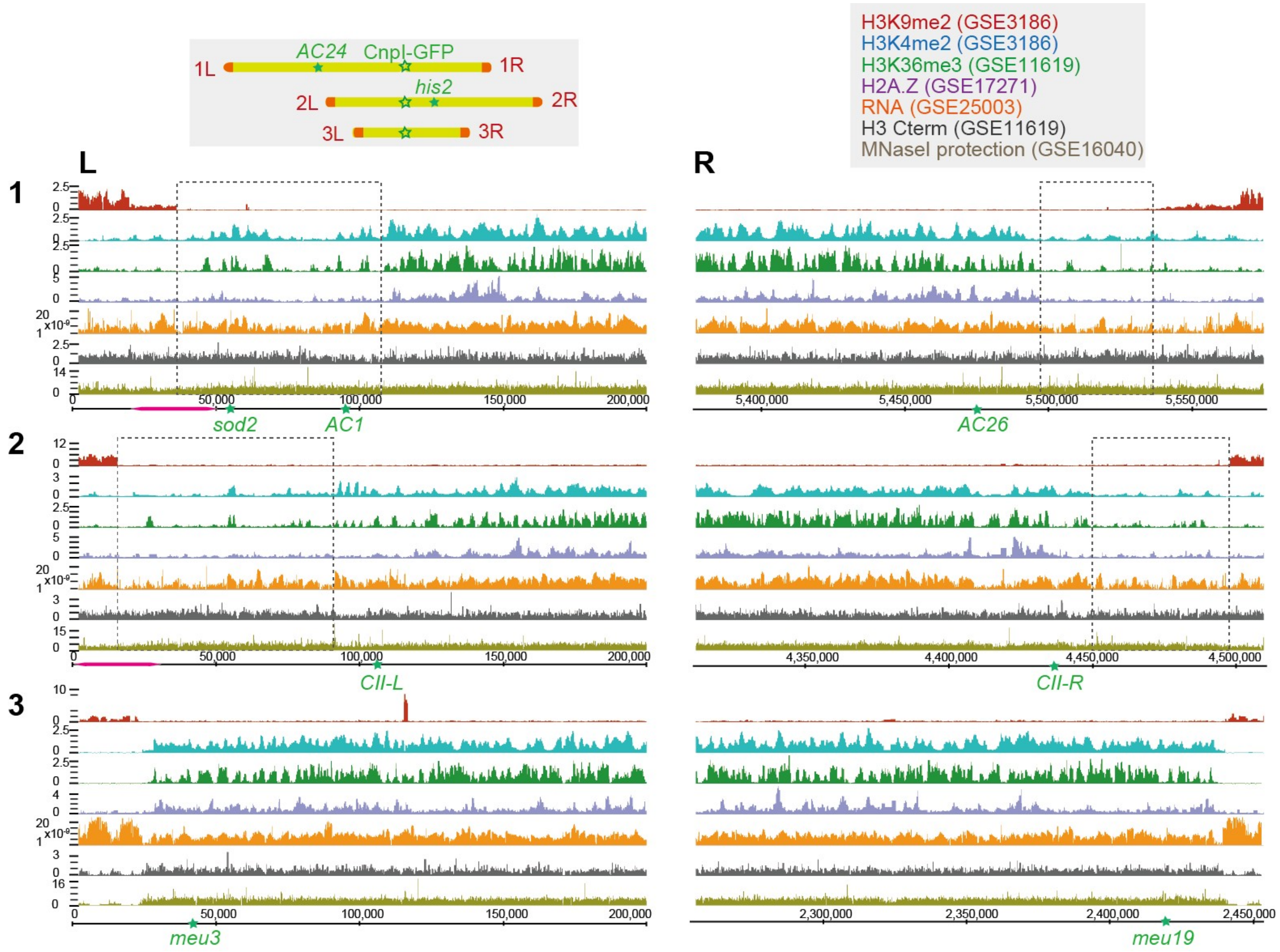

1L
AC24
CnpI-GFP
1R
his2
2L
2R
3L
3R
H3K9me2 (GSE3186)
H3K4me2 (GSE3186)
H3K36me3 (GSE11619)
H2A.Z (GSE17271)
RNA (GSE25003)
H3 Cterm (GSE11619)
MNaseI protection (GSE16040)
L
R
1
2
3
sod2
AC1
AC26
CII-L
CII-R
meu3
meu19

Supplementary Figure 5. A compiled summary of publicly available ChIP, RNA level or MNaseI protection data in the 200-kb regions from the ends of annotation

Top-left shows a map of all three chromosomes in *S. pombe*. The orange regions (200-kb from the end of annotation) are magnified and shown along with various data below. Color code for ChIP, RNA level and MNaseI protection is shown at the top-right with the corresponding accession numbers which the data were derived from. *LacO* insertion sites used in this study are indicated with filled green stars in the chromosome map and plots. Cnp1-GFP is present in all centromeres and indicated by open green stars. Within the plots, regions with hypomethylated H3 and with low RNA abundance are highlighted with gray-dotted boxes. Two regions in 1L and 2L with very high homology (99% identity) are underlined in pink.

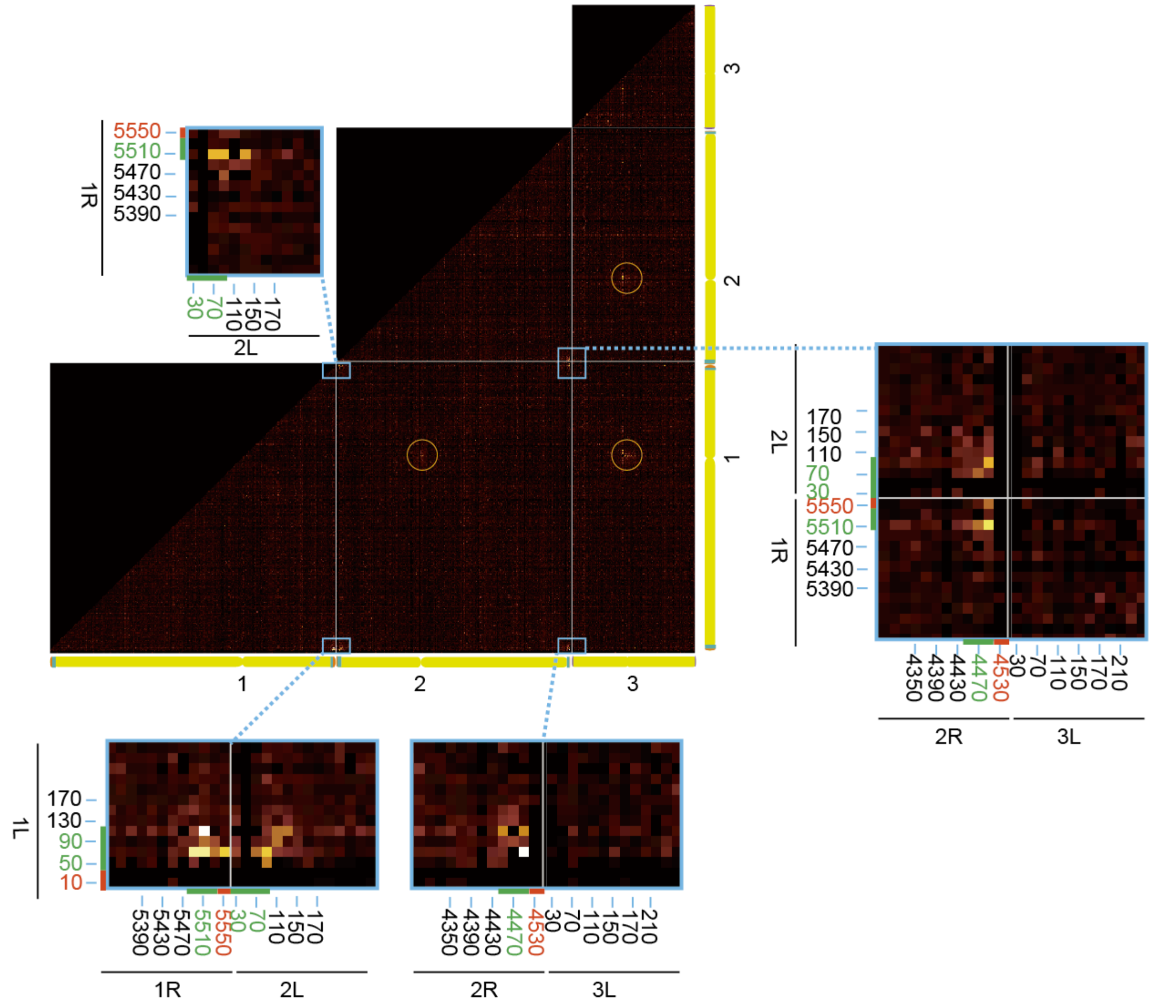

Supplementary Figure 6. Summary of Hi-C analysis

The distance-normalized physical proximity values (Tanizawa, H. *et al. Nucleic Acids Res.* **38,** 8164–8177 (2010)) are mapped along the genome coordinates. Yellow bars show the positions of the three chromosomes. Regions circled in orange show centromeric association and regions squared in blue show subtelomeric association. The regions of subtelomeric association were magnified. Coordinates are given in kilobases, and the colors represent euchromatin (black), silent regions (red), and ST-chromatin (green).

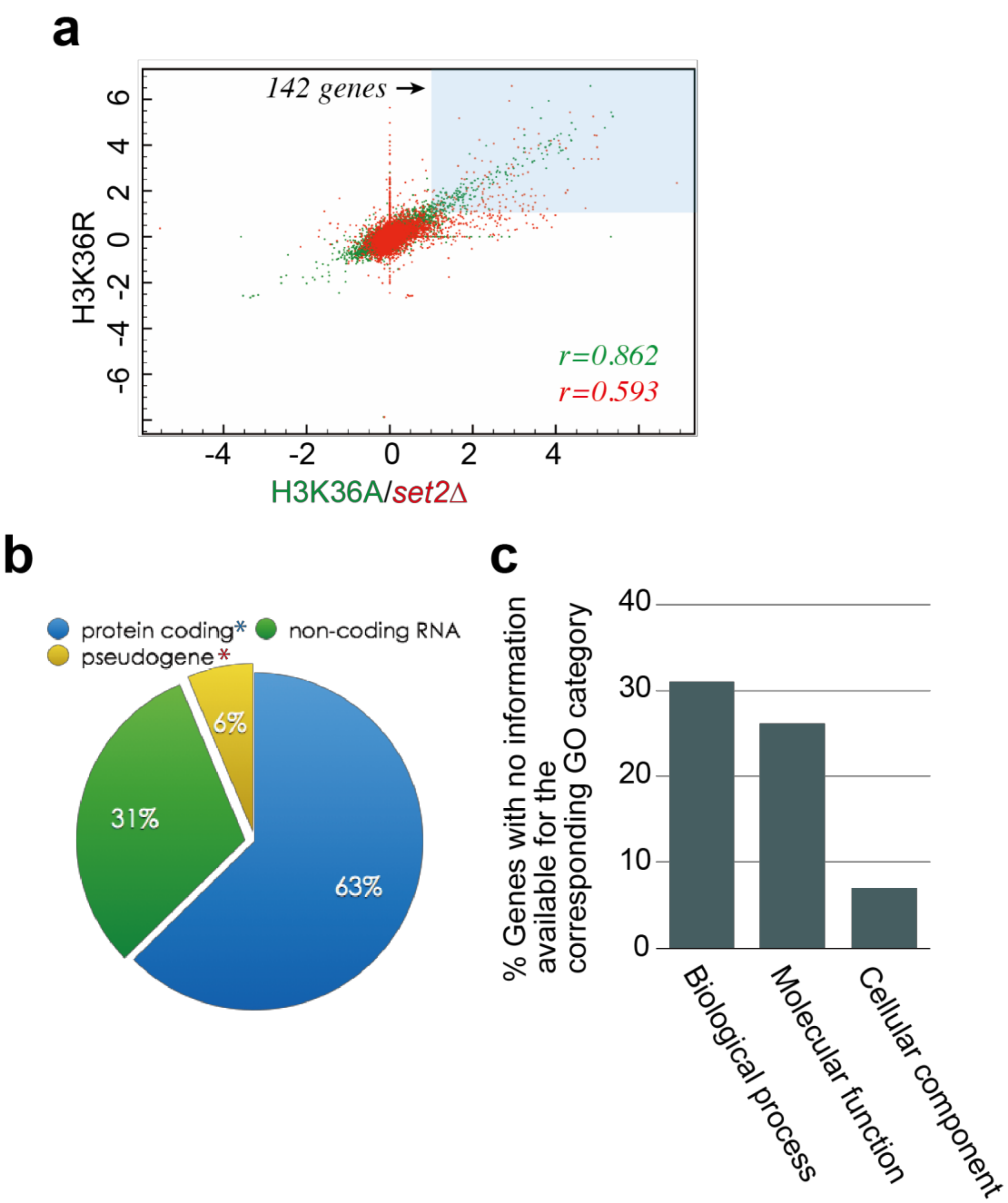

Supplementary Figure 7. A summary of the genes upregulated in *set2*Δ, H3K36R, and H3K36A

(a) A plot of the log2 ratio of gene expression in H3K36R compared with H3K36A (green) or *set2*Δ (red). Correlation coefficients (*r*) are shown in the plot in corresponding colors. No genes were found to be downregulated more than two-fold in all three strains, but 142 genes were upregulated more than two-fold in all three strains.

(b) All 142 upregulated genes were classified as “protein coding,” “non-coding RNA,” or “pseudogenes.” Classes identified at significantly higher (red) or lower (blue) frequency (chi-squire test, $p < 0.001$) are marked by asterisks. (c) Of the 89 protein-coding genes, the fraction for which a gene ontology (GO) term has not yet been assigned is shown. All three categories showed a significantly higher frequency than that expected by random selection (chi-square test, $p < 0.0002$).

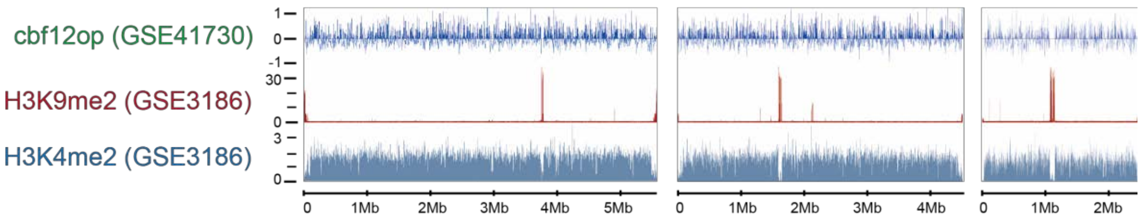

Supplementary Figure 8. Gene expression of cells overproducing Cbf12 mapped to chromosome coordinates

A plot of the log2 ratio of gene expression in cells overproducing Cbf12, shown with ChIP data for H3K9me2 and H3K4me2 for comparison. Accession numbers are shown in parentheses.

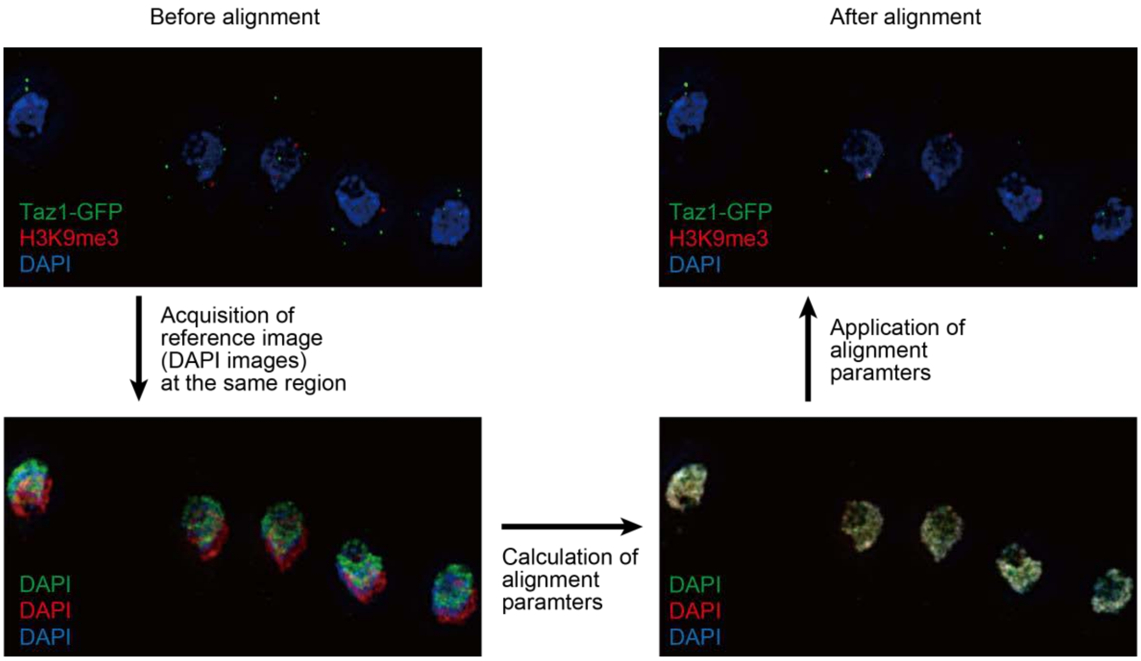

Supplementary Figure 9. An example of registration using the DAPI image as a reference

A merged image of all three channels, not yet aligned, is shown at the top left. After image acquisition, another set of images (bottom left) was acquired using the same DAPI image in all three channels. Alignment parameters were calculated using this image (bottom right). Finally, the same parameters were used to align the target image (top right).

Supplementary Table 1. Gene ontology of genes commonly upregulated in all *set2*Δ, H3K36R and H3K36A

**Biological process**		Number of genes assigned
	biological_process*	44
	cell adhesion*	5
	transmembrane transport*	4
	meiotic cell cycle	4
	pyruvate metabolic process*	3
	amino acid transmembrane transport*	3
	other (less than two genes were assigned)	53
**Molecular function**		
	molecular_function*	37
	metal ion binding	6
	ATP binding	5
	zinc ion binding	4
	transmembrane transporter activity*	4
	hydrolase activity*	4
	methyltransferase activity*	3
	amino acid transmembrane transporter activity*	3
	RNA polymerase II core promoter proximal region sequence-specific DNA binding	3
	other (less than two genes were assigned)	62
**Cellular component**		
	nucleus	31
	cytosol	29
	integral component of membrane*	28
	endoplasmic reticulum*	16
	external side of plasma membrane*	12
	cytoplasm	10

cellular_component*	10
plasma membrane*	7
cell surface*	5
fungal-type vacuole*	4
external side of cell wall*	4
Golgi apparatus	4
extracellular region*	3
anchored component of membrane*	3
other (less than two genes were assigned)	19

* Significantly higher frequency than random selection (chi-square test, $p<0.0002$).

Supplementary Table 2. Genes with nuclear localization (according to GO terms) and commonly upregulated more than twice in all *set2*Δ, H3K36R and H3K36A.

Chr	Start	Stop	GeneName	Description	*set2*Δ	H3 K36R	H3 K36A
chr1	1	5662	*tlh1*	RecQ type DNA helicase	3.59	3.99	3.85
chr1	27353	27763	SPAC212.02	Schizosaccharomyces pombe specific protein	4.10	4.05	3.88
chr1	32230	32670	SPAC977.02	S. pombe specific 5Tm protein family	3.78	3.76	4.13
chr1	*46075*	*48160*	SPAC977.09c	phospholipase (predicted)	1.05	1.63	1.65
chr1	*62989*	*63732*	SPAC977.15	dienelactone hydrolase family	1.44	1.74	1.51
chr1	*92480*	*93871*	SPAC1F8.04c	hydrolase (predicted)	2.26	1.51	1.58
chr1	153387	154535	SPAC5H10.04	NADPH dehydrogenase (predicted)	3.24	1.85	1.80
chr1	3160230	3161942	SPAC3G9.11c	pyruvate decarboxylase (predicted)	2.62	1.80	1.52
chr1	3826640	3828589	SPAC4H3.03c	glucan 1,4-alpha-glucosidase (predicted)	1.29	1.40	1.56
chr1	*5498273*	*5498965*	*pcm2*	protein-L-isoaspartate O-methyltransferase	3.10	3.91	4.10

				Pcm2 (predicted)			
chr1	*5517959*	*5519242*	SPAC869.02c	nitric oxide dioxygenase (predicted)	1.37	1.84	1.64
chr1	*5529962*	*5530960*	SPAC186.02c	hydroxyacid dehydrogenase (predicted)	3.10	4.76	5.18
chr1	*5538574*	*5539101*	SPAC186.04c	N-terminal of transmembrane channel, truncated	2.33	2.61	2.57
chr1	*5548884*	*5550012*	SPAC186.08c	L-lactate dehydrogenase (predicted)	3.00	2.67	3.10
chr1	5552811	5554529	SPAC186.09	pyruvate decarboxylase (predicted)	3.36	3.66	4.07
chr2	12176	12613	SPBC1348.04	methyltransferase (predicted)	2.59	2.32	2.42
chr2	*35112*	*36589*	SPBC1348.12	transcription factor (predicted)	3.24	4.69	4.47
chr2	*49143*	*51181*	*grt1*	transcription factor Grt1 (predicted)	2.48	2.29	1.78
chr2	128825	129598	*mug14*	adducin	1.33	1.21	1.71
chr2	454230	456312	*meu6*	meiotic chromosome segregation protein Meu6	3.34	1.36	1.01
chr2	2660490	2660921	*hsp16*	heat shock protein Hsp16	1.04	1.96	1.22

chr2	*4453078*	*4453803*	SPBP4G3.03	PI31 proteasome regulator (predicted)	3.33	2.82	2.65
chr2	*4466754*	*4467515*	SPBPB2B2.05	peptidase family C26 protein	2.37	1.66	1.92
chr2	*4469200*	*4471005*	SPBPB2B2.06c	phosphoprotein phosphatase (predicted)	6.92	2.34	1.96
chr2	*4476012*	*4476674*	SPBPB2B2.08	conserved fungal protein	4.86	3.40	3.31
chr2	*4484059*	*4485156*	SPBPB2B2.11	nucleotide-sugar 4,6-dehydratase (predicted)	1.21	1.14	1.21
chr2	*4485277*	*4487418*	*gal10*	UDP-glucose 4-epimerase/aldose 1-epimerase Gal10	3.20	1.80	1.94
chr2	4501956	4502396	SPBPB2B2.17c	S. pombe specific 5Tm protein family	3.78	3.76	4.13
chr2	4526885	4532644	*tlh2*	RecQ type DNA helicase Tlh1	3.59	3.99	3.85
chr3	1699682	1701301	SPCC417.12	lisophospholipase family protein	2.97	1.74	1.41
chr3	1871750	1874641	*cbf12*	CBF1/Su(H)/LAG-1 family transcription factor Cbf12	2.09	1.12	1.02

Positions with underlines and in italic indicate subtelomeric silent regions and knob regions, respectively.

Supplementary Table 3 *S. pombe* strains used in this study

Strain	Genotype
972	h^{-}
968	h^{90}
YAM018	h^{-} lys1^{+}::(hta1 htb1-GFP)
YAM016	h^{-} lys1^{+}::(hta1 GFP-htb1)
YAM029	h^{90} ade6-216 lys1^{+}::(hhf2 hht2-GFP)
TGO160	h^{90} ade6-26 leu1 ura4 GFP-hht2
HMP5	h^{-} hht1^{+}-GFP-kan^{r}
HMP1611	h^{-} cnp1^{+}-yeGFP-kan^{r}
YAM055	h^{-} taz1^{+}-GFP-kan^{r}
CT2121-4	h^{90} ade6-216 leu1 lys1 ura4 sod2.proxy::kan^{r}-ura4^{+}-lacO his7^{+}::lacI-GFP
YY705-2A	h^{90} ade6-216 leu1 lys1 ura4 AC1::ura4^{+}-kan^{r}-lacO his7^{+}::lacI-GFP
YY695-1	h^{90} ade6-216 leu1 lys1 ura4 mbs1::kan^{r}-ura4^{+}-lacO aur1-r::lacI-GFP
YY774-4	h^{90} ade6-216 leu1 lys1 ura4 AC24::ura4^{+}-kan^{r}-lacO his7^{+}::lacI-GFP
YY775-1	h^{90} ade6-216 leu1 lys1 ura4 AC26::ura4^{+}-kan^{r}-lacO his7^{+}::lacI-GFP
YW069-1	h^{90} ade6-216 leu1 lys1 ura4 CII-L::kanr-ura4^{+}::lacO his7^{+}::lacI-GFP
MK153	h^{-} ade6-M216 leu1 lys1 ura4 his2::kan^{r}-ura4^{+}-lacO his7^{+}::lacI-GFP
YW071-2	h^{90} ade6-216 leu1 lys1 ura4 CII-R::kan^{r}-ura4^{+}::lacO his7^{+}::lacI-GFP
YY964-2	h^{90} ade6-216 leu1 lys1 ura4 meu3::kan^{r}-ura4^{+}-lacO his7^{+}::lacI-GFP
YY960-2	h^{90} ade6-216 leu1 lys1 ura4 meu9::kan^{r}-ura4^{+}-lacO his7^{+}::lacI-GFP
HR236	h^{-} cdc10-129
HR389	h^{+} cdc25-22
RK5	h^{-} clr3::kan^{r}

YAM064	h^- *dcr1::hph*
YAM059	h^- *clr4::hph*
YAM061	h^- *swi6::hph*
YAM071	h^- *set9::kan*^{r}
YAM065	h^- *set1::kan*^{r}
YAM070	h^- *msc1::hph*
YAM074	h^{90} *msc1::kan*^{r}
YAM040	h^- *set2::kan*^{r}
YAM073	h^{90} *set2::kan*^{r}
HA1050-5B	h^{90} *leu1 lys1 ura4 (hht1 hhf1):: kan*^{r} *(hht3 hhf3)::NAT*
YAM233	h^{90} *leu1 lys1 ura4 (hht1 hhf1):: kan*^{r} *hht2(K36R) (hht3 hhf3)::NAT*
YAM232	h^{90} *leu1 lys1 ura4 (hht1 hhf1):: kan*^{r} *hht2(K36A) (hht3 hhf3)::NAT*
YAM283	h^{90} *leu1 lys1 ura4 (hht1 hhf1):: kan*^{r} *hht2(K36R) (hht3 hhf3)::NAT set2::hph*
YAM284	h^{90} *leu1 lys1 ura4 (hht1 hhf1):: kan*^{r} *hht2(K36R) (hht3 hhf3)::NAT set2::hph*
YAM260	h^{90} *leu1 lys1 ura4 (hht1 hhf1):: kan*^{r} *hht2(K36Q) (hht3 hhf3)::NAT*
YAM101	h^+ *his2 leu1 lys1 ura4 cdc10-129*
YAM204	h^+ *his2 leu1 ura4 cdc10-129 clr6-1*
YAM253	h^+ *his2 leu1 lys1 ura4 cdc10-129 set2::kan*^{r}
YAM255	h^+ *his2 leu1 ura4 cdc10-129 clr6-1 set2::kan*^{r}
YAM113	h^- *leu1 lys1 ura4 1R1::ura4*$^+$
YAM114	h^- *leu1 lys1 ura4 1R2::ura4*$^+$
YAM115	h^- *leu1 lys1 ura4 1R3::ura4*$^+$
YAM116	h^- *leu1 lys1 ura4 1R4::ura4*$^+$

YAM117	*h⁻ leu1 lys1 ura4 1R5::ura4⁺*
YAM118	*h⁻ leu1 lys1 ura4 1R6::ura4⁺*
YAM119	*h⁻ leu1 lys1 ura4 1R7::ura4⁺*
YAM120	*h⁻ leu1 lys1 ura4 1R8::ura4⁺*
YAM121	*h⁻ leu1 lys1 ura4 1R9::ura4⁺*
YAM122	*h⁻ leu1 lys1 ura4 1R10::ura4⁺*
YAM153	*h⁻ leu1 lys1 ura4 1R11::ura4⁺*
YAM211	*h⁻ leu1 lys1*
YAM212	*h⁻ leu1 lys1 ura4*
YAM175	*h⁻ leu1 lys1 ura4 dg1L::ura4⁺*
YAM187	*h⁺ leu1 lys1 ura4 matP::ura4⁺*
YAM123	*h⁻ leu1 lys1 ura4 1R1::ura4⁺ swi6::hph*
YAM124	*h⁻ leu1 lys1 ura4 1R2::ura4⁺ swi6::hph*
YAM125	*h⁻ leu1 lys1 ura4 1R3::ura4⁺ swi6::hph*
YAM126	*h⁻ leu1 lys1 ura4 1R4::ura4⁺ swi6::hph*
YAM127	*h⁻ leu1 lys1 ura4 1R5::ura4⁺ swi6::hph*
YAM128	*h⁻ leu1 lys1 ura4 1R6::ura4⁺ swi6::hph*
YAM129	*h⁺ his2 leu1 lys1 ura4 1R7::ura4⁺ swi6::hph*
YAM130	*h⁻ leu1 lys1 ura4 1R8::ura4⁺ swi6::hph*
YAM131	*h⁻ leu1 lys1 ura4 1R9::ura4⁺ swi6::hph*
YAM132	*h⁺ his2 leu1 lys1 ura4 1R10::ura4⁺ swi6::hph*
YAM154	*h⁻ leu1 lys1 ura4 1R11::ura4⁺ swi6::hph*
YAM164	*h⁻ leu1 lys1 swi6::hph*
YAM161	*h⁻ leu1 lys1 ura4 swi6::hph*

YAM172	*h*$^{-}$ *leu1 lys1 ura4 dg1L::ura4*$^{+}$ *swi6::hph*
YAM201	*h*$^{+}$ *leu1 lys1 ura4 matP::ura4*$^{+}$ *swi6::hph*
YAM133	*h*$^{-}$ *leu1 lys1 ura4 1R1::ura4*$^{+}$ *set2::kan*^{r}
YAM134	*h*$^{-}$ *leu1 lys1 ura4 1R2::ura4*$^{+}$ *set2::kan*^{r}
YAM135	*h*$^{-}$ *leu1 lys1 ura4 1R3::ura4*$^{+}$ *set2::kan*^{r}
YAM136	*h*$^{-}$ *leu1 lys1 ura4 1R4::ura4*$^{+}$ *set2::kan*^{r}
YAM137	*h*$^{-}$ *leu1 lys1 ura4 1R5::ura4*$^{+}$ *set2::kan*^{r}
YAM138	*h*$^{-}$ *leu1 lys1 ura4 1R6::ura4*$^{+}$ *set2::kan*^{r}
YAM139	*h*$^{+}$ *his2 leu1 lys1 ura4 1R7::ura4*$^{+}$ *set2::kan*^{r}
YAM140	*h*$^{-}$ *leu1 lys1 ura4 1R8::ura4*$^{+}$ *set2::kan*^{r}
YAM141	*h*$^{-}$ *leu1 lys1 ura4 1R9::ura4*$^{+}$ *set2::kan*^{r}
YAM142	*h*$^{-}$ *leu1 lys1 ura4 1R10::ura4*$^{+}$ *set2::kan*^{r}
YAM155	*h*$^{-}$ *leu1 lys1 ura4 1R11::ura4*$^{+}$ *set2::kan*^{r}
YAM163	*h*$^{-}$ *leu1 lys1 set2::kan*^{r}
YAM160	*h*$^{-}$ *leu1 lys1 ura4 set2::kan*^{r}
YAM176	*h*$^{-}$ *leu1 lys1 ura4 dg1L::ura4*$^{+}$ *set2::kan*^{r}
YAM189	*h*$^{+}$ *leu1 lys1 ura4 matP::ura4*$^{+}$ *set2::kan*^{r}
YAM202	*h*$^{-}$ *leu1 lys1 set2::kan*^{r} *taz1-GFP::hph*
YAM203	*h*$^{+}$ *leu1 lys1 swi6::hph taz1-GFP::kan*^{r}

The approximate positions of the *lacO* insertions of genome version 2004 Sep are as follows:

sod2.proxy=ch1:50,946, *AC1*=ch1:94,500, *mbs1*=ch1:765,526, *AC24*=ch1:1,866,360,

AC26=ch1:5,479,451, *CII-L*=ch2:103,000, *CII-R*=ch2:4,404,000.

The exact positions of *ura4* insertions in chromosome 1 of genome version 2004 Sep are as follows: *1R1*=5,578,599, *1R2*=5,570,000, *1R3*=5,559,919, *1R4*=5,550,290, *1R5*=5,539,787, *1R6*=5,529,557, *1R7*=5,520,377, *1R8*=5,510,098, *1R9*=5,499,698, *1R10*=5,486,769, *1R11*=5,479,500 (*AC26*). *dg1L* is in the dg repeat of the left arm of chromosome 1 at the

position between primer annealing sequences “5’-CGACCAATATGCTGCGGTTCACC” and “5’-CTTAACATCATGTTTTTAACCAACGAC”. *matP* is close to the *matP* locus at the position between primer annealing sequences “5’-GAAGTACTGCTATCCCCAAATTGG” and “5’-CTTACGCTTCCTTGTTAGTCCGTTAG”.

Supplementary Table 4 Antibodies used in this study

Epitope	Antibody name	Source	Concentration used	Supplier
H3K4Ac	39382	Rabbit	1/500 dilution	Active Motif (Carlsbad, USA)
H3K4 (unmodified)		Mouse	4 μg ml^{-1}	[1]
H3K4me1	CMA302(19A5)	Mouse	4 μg ml^{-1}	[1]
H3K4me2	CMA303(27A6)	Mouse	4 μg ml^{-1}	[1]
H3K4me3	CMA304(16H10)	Mouse	4 μg ml^{-1}	[1]
H3K9Ac	CMA310(19E5)	Mouse	4 μg ml^{-1}	[2]
H3K9 (unmodified)	1C6	Mouse	4 μg ml^{-1}	*
H3K9me1	CMA316(2B8)	Mouse	16 μg ml^{-1}	[2]
H3K9me2	CMA317(6D11)	Mouse	4 μg ml^{-1}	[2]
H3K9me3	CMA318(2F3)	Mouse	4 μg ml^{-1}	[2]
H3K36me2	CMA332(2C3)	Mouse	4 μg ml^{-1}	[3]
H3K36me3	CMA333(13C9)	Mouse	4 μg ml^{-1}	[3]
H4K20me1	22G3	Mouse	4 μg ml^{-1}	[4]
H4K20me2	2E2	Mouse	4 μg ml^{-1}	[4]
H4K20me3	27F10	Mouse	4 μg ml^{-1}	[4]
H3S10Sph	CMA311	Mouse	4 μg ml^{-1}	[5]
RNAPII (unmodified)	CMA601	Mouse	4 μg ml^{-1}	[6]
RNAPIIser5ph	CMA603	Mouse	4 μg ml^{-1}	[6]

RNAPIIser2ph	CMA602	Mouse	4 μg ml^{-1}	[6]
GFP	598	Rabbit	1/500 dilution	MBL (Nagoya, Japan)
GFP	GFP-booster Atto488	Llama	5 μg ml^{-1}	ChromoTeck (Martinsried, Germany)
Mouse IgG	A11017 (Alexa Fluor 488)	Goat	1/500 dilution	Invitrogen (Carlsbad, USA)
Mouse IgG	A11031 (Alexa Fluor 568)	Goat	1/500 dilution	Invitrogen (Carlsbad, USA)
Rabbit IgG	A21206 (Alexa Fluor 488)	Donkey	1/500 dilution	Invitrogen (Carlsbad, USA)
Rabbit IgG	A11034 (Alexa Fluor 568)	Goat	1/500 dilution	Invitrogen (Carlsbad, USA)

Supplementary References

1. Kimura, H., Hayashi-Takanaka, Y., Goto, Y., Takizawa, N. & Nozaki, N. The Organization of Histone H3 Modifications as Revealed by a Panel of Specific Monoclonal Antibodies. *Cell Struct. Funct.* **33,** 61–73 (2008).

2. Hayashi-Takanaka, Y. *et al.* Tracking epigenetic histone modifications in single cells using Fab-based live endogenous modification labeling. *Nucleic Acids Res.* **39,** 6475–6488 (2011).

3. Rechtsteiner, A. *et al.* The Histone H3K36 Methyltransferase MES-4 Acts Epigenetically to Transmit the Memory of Germline Gene Expression to Progeny. *PLoS Genet* **6,** e1001091 (2010).

4. Eberhart, A. *et al.* Epigenetics of eu- and heterochromatin in inverted and conventional nuclei from mouse retina. *Chromosome Res.* **21,** 535–554 (2013).

5. Hayashi-Takanaka, Y., Yamagata, K., Nozaki, N. & Kimura, H. Visualizing histone modifications in living cells: spatiotemporal dynamics of H3 phosphorylation during interphase. *J. Cell Biol.* **187,** 781–790 (2009).

6. Stasevich, T. J. *et al.* Regulation of RNA polymerase II activation by histone acetylation in single living cells. *Nature* **515,** 272-275 (2014).

* Kimura et al., unpublished results.
